# Closing Water and Nutrient Cycles in Urban Wastewater Management: How to Make an Academic Software Available to General Practice

**DOI:** 10.1007/s43615-021-00073-6

**Published:** 2021-07-15

**Authors:** Johann S. Schuur, Dorothee Spuhler

**Affiliations:** 1 Eawag, Swiss Federal Institute of Aquatic Science and Technology, 8600 Dübendorf, Switzerland; 2 Institute of Science, Technology and Policy, ETH Zürich, 8092 Zürich, Switzerland

**Keywords:** Circular economy, Sustainable sanitation, Resource recovery, Human-centred design, Personas, Wastewater technologies

## Abstract

Appropriate sanitation is crucial to alleviate pressures on environmental and human health hazards. Conventional (sewered) sanitation systems are often not viable in rapidly developing urban areas, where over 70% of the world population is expected to live in 2050. Freshwater is polluted and valuable resources such as nutrients and organics are lost. At present, many alternative sanitation technologies and systems are being developed with the aim to alleviate these pressures through (1) independency from sewers, water, and energy, therefore better adapted to the needs of fast and uncontrolled developing urban areas; and (2) contribute to a circular economy through the recovery of nutrients, energy, and water for reuse. Unfortunately, these innovations hardly find their way into practice because there exists a lack of data and knowledge to systematically consider them in strategic planning processes. To this end, we have developed SANitaTIon system Alternative GeneratOr (SANTIAGO)—a software that provides a comprehensive list of potential technologies and system configurations and quantifies their local appropriateness as well as their resource recovery and loss potentials. The aim is to provide a manageable but diverse set of decision options together with information needed to rank the alternatives and to select the preferred one in a structured decision making process. To make this software useful for practice, an easily accessible interactive user interface is required that (1) facilitates data collection and input; and (2) the exploration and presentation of results. As a first step in creating this user interface, we develop a framework that summarizes (1) the requirements that arise from practical applications of SANTIAGO, and (2) a comprehensive user understanding on the basis of 21 interviews with international practitioners caught in five personas: capacity developers, engineering experts, planners, researchers, teachers and trainers. This framework aids the development of any academic software into a tool useful for practice and policy makers. Here specifically, it enables contribution to sustainable development goals 6 (clean water and sanitation), and 11 (sustainable cities and communities).

**Supplementary Information:**

The online version contains supplementary material available at 10.1007/s43615-021-00073-6.

## Introduction

The worlds’ population reached 7.7 billion people in 2019, is expected to increase to 9.7 billion in 2050, and virtually all of this growth is likely to be absorbed in urban areas [1]. Cities already hosted 50% of the global population in 2008 and this is predicted to grow close to 70% in 2050 [2, 3]. Where Europe and Northern America are stabilizing, tremendous growth is seen in the global south, notably the urban population of Africa and Asia that is predicted to double in less than one generation [1, 4, 5]. In places where growth is uncontrolled and rapid (informal settlements, slums, and emerging small towns), it often leads to issues such as environmental degradation, severe public health hazards as well as pressures on valuable resources like water and nutrients [6]. These issues are exemplified by a lack of accessibility to safely managed sanitation^1^, a human right available to only 45% of the global population in 2017 [7]. Moreover, the wastewater from more than half of the population is thus not disposed of safely and is subsequently released into the environment without appropriate treatment [7, 8]. To achieve access for all to even basic sanitation services by 2030, a doubling of the current annual rate of progress is required [9].

The availability, development, and planning of effective and sustainable sanitation systems are therefore key in the support of sustainable urban growth. Sustainable sanitation systems not only protect the human health and the environment, they are also economically viable, socially acceptable, technically and institutionally appropriate, and close water and nutrient cycles at the lowest possible level in order to recover resources and protect people downstream. The importance of sustainable sanitation is further stressed by the adoption of “Clean water and sanitation” as one of the sustainable development goals [9]. The problem till date however is that there exists a large variety in available resources, knowledge, spatial possibilities, maintenance and operation across the world. For example, in fast-growing areas of developing countries, high density, informality, lack of administrative and financial resources for planning, implementation, and operation of safe sanitation intensify the present issues [2, 10, 11]. Yet, funding requirements often lead to the adoption of conventional “one size fits all” sewered and centralized approaches. Where in some areas these solutions seem effective, the peculiarities of another context often result in system failure and waste of funding [12–14].

For these reasons, it is crucial that sanitation technologies are selected that prove appropriate in a specific context. At present, there are over 50 technologies available and more are being developed [15–19]. Many are suitable to be implemented on a decentralized basis, independent from sewers, energy or even water. In addition, they are, compared to centralized systems, more flexible to adapt and thereby respond appropriately to changing socio-demographic and environmental conditions. Moreover, they allow for the recovery of nutrients, energy and water which can in turn be used for agriculture or urban green infrastructure thereby help paving the way to a circular economy. This has triggered the development of many novel sanitation technologies and system configurations and options for faecal sludge management (FSM) both as alternatives to the sewered centralised solution. Examples include urine diversion dry toilets or container-based sanitation [20, 21]. However, their uptake in practice remains slow because of a number of reasons. First, many are still unknown with the wider public and there exists unwillingness to adopt some technologies because they lack the backing from large donor organizations. This is mainly because new technologies mis centuries of knowledge, and performance data that does exists for conventional sewer systems. Also, as compared to centralized systems, different operation and maintenance, and service provision models have to be adopted in collaboration with the private sector. Second, combining over 50 technologies into sanitation systems results in more than 100.000 decision options which pose an almost impossible challenge for even the most experienced planner. The planning complexity, as compared to conventional solutions, is further enhanced by an ever-growing number of technology options and criteria, let alone the trade-offs that arise and often conflicting stakeholder preferences.

### State of the Art

These challenges are continuously being addressed by theoretical well-planned approaches such as the *Compendium of Sanitation Options and Technologies* and a multitude of other specialized tools and approaches [15, 22, 23]. Spuhler and Lüthi however, indicated that there remains a lack of systematic tools to identify suitable sanitation technologies and systems from the multiplicity of options that exist today [24]. And, despite the availability of such a rich suit of specialized tools and approaches as well as the continuous development of their theoretical foundation, many limitations are known, they are difficult to use for non-experts, and as a result rarely used in practice [23, 25–27]. Moreover, within capacity development, many manuals incorporate European and US standards, making them ill-suited for contexts where the need for appropriate sanitation solutions might be higher [28, 29].

The complexity of implementing sanitation systems—as crucial component of the WASH infrastructure delivery—goes beyond technical considerations alone and led to a call for more systems thinking tools that would bring understanding to this complexity. Specifically, “Systems tools can be useful for provoking discussions, aligning perspectives, identifying leverage points, designing interventions, or evaluating project outcomes” [30]. In subsequent work, Valcourt et al. argue that there is additionally an inadequate level of information—from existing approaches—to evaluate the utility and efficacy of system approaches. Increasing this level would require the evaluation of interconnections between factors (system elements); an expansion of geopolitical application; improved reporting of required resources for implementation; and increasing transparency of the outcomes of systems approaches [31].

**Fig. 1:**
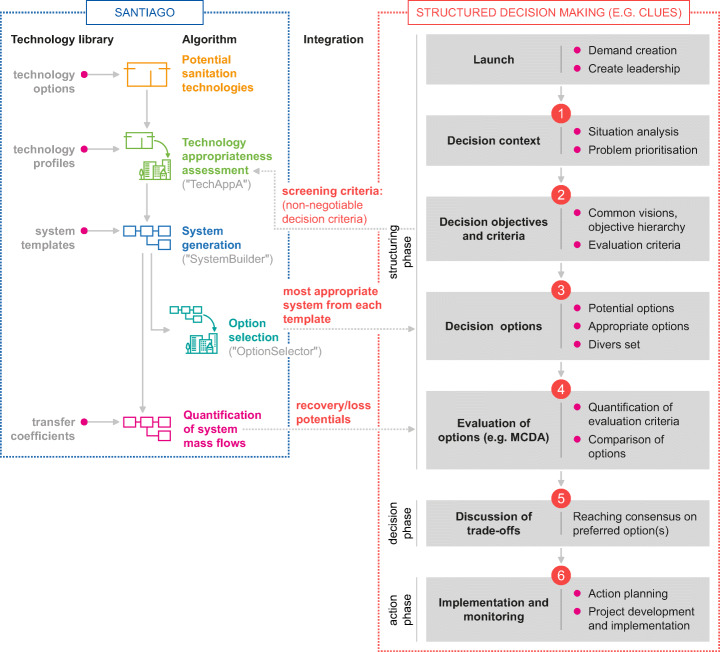
Reprinted with permission from [32]. Integration of SANTIAGO with a Structured Decision Making approach. Inputs for the software are decision objectives used to derive screening criteria. These arise from participatory stakeholder workshops and allow assessment of the appropriateness of potential sanitation technologies for a specifc context. The software comprises a technology library that identifies potential technologies. The output of SANTIAGO then consists of all possible system configurations, an appropriateness scores to assess their context-specific suitability, and a quantifies resource recovery potential as well as environmental emissions, and the most appropriate option or system. This then forms the input to be handed over for further evaluation in the decision-making process

These needs signal a contemporary gap between research and practice. To address this gap, we are continuously developing the SANiTatIon system option GeneratOr (SANTIAGO) (see Fig. 1) [32]. SANTIAGO is a generic software that enables the automated consideration of a large and diverse range of conventional and novel technologies on the basis of a set of local appropriate criteria that can be matched to a specific context. Furthermore, it assists in generating context-specific sanitation system configurations from single technologies and quantifies their resource recovery as well as resource loss potential.

At present, the software comprises four algorithms and a technology library that support the user in (1) evaluating the appropriateness of a set of potential technologies considering multiple criteria; (2) generating all possible sanitation system configurations; (3) selecting a set of systems which is diverse enough to highlight trade-offs among different decision objectives (e.g. costs and resource recovery) yet remains of manageable size; and (4) quantifying potential resource recovery as well as losses. The latter at present considers phosphorous and nitrogen (fertilizer and/or polluter), total solids (as indicator for energy potential and organics), and water (scarce and crucial for e.g. drinking water and agriculture). The resulting set of sanitation options forms the input for a more detailed evaluation in a participative strategic planning process e.g. multi-criteria analysis [33].

SANTIAGO is flexible to be applied for any (future) technology or any case at hand. And it systematically considers uncertainties to be applicable at an early planning phase also for very novel technologies. Six practical applications in Nepal, Ethiopia, Peru, and South Africa, revealed that SANTIAGO brings a number of advantages [32]. First, it allows for a systematic evaluation of local appropriateness of technologies based on socio-economic acceptance and technical feasibility. Thereby enhancing transparency of the selection process. Second, it enables the consideration of novel technologies and system configurations beyond the scope of prior experience. Thirdly, it enforces a system approach by selecting only valid system configurations within the entire sanitation value chain. Fourth, it allows to matches international knowledge and data to local conditions allowing more empirical decision making thereby enhancing reproducibility. Fifth, it allows to quantitatively compare the resource efficiency of all options. And last, it uses a multi-criteria decision analysis approach to enable the negotiation of trade-offs and find options that are agreed on by all stakeholders. SANTIAGO thus has the potential to enable practitioners and researchers to prioritise locally appropriate and resource efficient sanitation solutions at an early stage. Specifically, this helps to ex-ante devise sanitation solutions that avoid environmental degradation, public health hazards, and minimize losses of valuable resources thereby contributing to a circular and sustainable economy.

To make this software useful for practice, an easily accessible interactive user interface is required that facilitates (1) data collection and input; and (2) the exploration and presentation of results together with stakeholders. The software together with the user interface could become a tool that can be used to support decision making in planning, as well as for training and awareness raising on the diversity of technologies and systems and criteria for appropriate and sustainable sanitation. As a planning tool, it makes international expert and literature data available on a broad range of sanitation technologies and systems, helps to transparently identifying a set of locally appropriate options to be considered by the decision making process, and quantifies important sustainability criteria such as resource recovery and losses. As awareness raising and training tool, it helps to sensitise people regarding the diversity of technical options that are currently available, and what and how to consider when selecting an option for a specific context. In order to reach this goal, the first step is to synthesize the results from using SANTIAGO in past applications and how these might be used for the prioritisation of resource efficient sanitation solutions. The second and third step, the focus of this paper, is to (1) create a fundamental understanding of expectations from practitioners in order to (2) develop a tool that is fit for uptake in practice. These steps lead to the following questions that form the specific aims of this research.

1. How can past applications of SANTIAGO help to guide the selection of more appropriate, resource efficient and circular sanitation systems?
2. How can we make SANTIAGO available to practitioners in order to promote its application so that the planning of more sustainable and circular sanitation solutions is enabled?
  - What would a broader public of practitioners expect from a SANTIAGO application
  - How can we synthesize these expectations to guide the development of an academic software into a design useful for practice?

These should lead to a fundamental understanding of the potential contributions of SANTIAGO to practice and of the experiences, requirements, key challenges, behaviours, and motivations of future users. Users include capacity builders, engineering experts, planners, researchers, and trainers. This understanding should help to guide the development of an open-source online SANTIAGO user interface—SaniChoice: a training and decision support tool for sanitation technology selection—and simultaneous that of any other decision support and training tool in the field of environmental management and sustainability.

## Methods

### Potential of SANTIAGO for Practice

To answer the first question, we use the results from example applications and several case studies (two in Nepal, two in Ethiopia, one in South Africa, and one in Peru) published in previous publications and summarise the findings that are relevant for practice. The theoretical description of SANTIAGO including example applications are presented in [19, 32, 34]. The experiences of the practical application in Ethiopia amended with the experiences from Nepal are presented in [32, 35, 36]. The practical applications were implemented with local partners from development agencies (e.g. Helvetas Swiss Intercooperation), local non-governmental organisations (e.g. Environmental and Public Health Organisation, ENPHO, Nepal), local consultants (e.g. 500B Solutions, Nepal), local research institutes (e.g. Arba Minch University, Ethiopia), and local governments (e.g. Arba Minch Town Municipality, Ethiopa). The example applications and the practical applications showed that the methods have several advantages over existing methods and have the potential to contribute to more structured and strategic decision making and beyond [32]. These advantages and contributions will be summarized in the result section.

### Design Requirements for SaniChoice

To answer the second question, we combine approaches that originate in human-centred design (development of personas) and user-experience design (user jobs, pains, and gains). Personas are a tool frequently adopted in human-centred design. They focus on the extraction of fundamental needs and desires from a future user in order to develop effective designs for products, tools, and experiences [37]. They elicit a thorough picture of the experiences, requirements, key challenges, behaviours and motivations of users in the field. They support empathy (internalize goals, needs and wants of the user), focus (who will use the platform, who will not), communication and consensus building (understanding the scope of users helps to build consensus on important matters) and help to make and defend design decisions. The goal is to gain a thorough understanding of participants and their perspectives, build empathy with how they see life and how they do their daily job. Particularly, the issues that our product, SaniChoice, will address. In the end, we want to be able to put ourselves into the users’ shoes so that we are able to develop a product that works for them (Fritz Brugger, personal communication, August 11, 2020). A general rule of thumb is that the number of people on which each persona is based is defined by the point of diminishing marginal returns (understanding). Personas are not the same as a role, because different behaviours can occur within one role. Therefore, usually two personas are needed per role.

User-experience design (UX-design) helps to understand specific pains and gains in the user experience and as a result allow us to design services that function as pain relievers and gain creators. The former is affected by seven factors as presented in the *UX-Honeycomb* [38], the latter can be explained by a *Value Propositions Canvas* (after Alexander Osterwalder).

The *UX-Honeycomb* allows the designer to balance and make explicit trade-offs between the following factors: *Useful*—The customer should be able to get the job done (practical aspect), and it should be fun and aesthetical pleasing to do so (non-practical aspect); *Usable*—enabling the user to effectively and efficiently achieve her objective; *Findable*—it should be easy to find the explicit content that the user requires without being confronted with content that is non-relevant; *Credible*—ability to trust the (use of) the product over a reasonable amount of time even when content is updated; *Desirability*—covering the branding, image, identity, aesthetics, and emotional design. Best case scenario is when a user promotes the platform to her peers; *Accessibility*—it should be accessible to users over the full range of abilities, for example to those that are vision impaired; *Valuable*—it is valuable to the customer when the ultimate combination and balance of the aforementioned factors is achieved.

The general order of questions should follow the *Why* (motivations for adopting the product; relation to the user task; values and views associated with ownership and use of the product), *What* (what can the user do with the product; what are the specific functionalities and features), and *How* (functionality in an accessible and aesthetically pleasing way).

We combine both human-centred design and UX design into a set of personas that help to focus on a manageable and memorable cast of fictional characters while designing the user interface. These personas help us to answer the second aim of this research and are constructed by following a specific roadmap:

Step 1: *Identification of potential users and sample personas*. An online tool is in theory accessible to everyone. To limit the scope of our research however, we first define five potential user groups: *capacity builders, engineering experts*, *planners*, *researchers*, *trainers and teachers*. We define a *capacity builder* as a person working in a capacity building or development agency as well as in institutes and foundations and is involved in the organizational development or backing of tool development, awareness raising, planning and training of people that work on sanitary solutions for urban settlements (in developing countries) on a management level. An *engineering expert* is defined as a wastewater or sanitation system expert working in research and development or a consultancy firm and is employed by any institution to analyse, develop and give advice on wastewater technologies in urban settlements. *Planners* are defined as (governmental) officials in charge of developing (new) urban wastewater management schemes. We depict *researchers* as those working in the field of sustainable wastewater technologies, policy development and social sciences to review, improve or develop novel and current sanitation solutions. We combine *teachers and trainers* into one persona because we expect their needs to be similar. We define this category of users as anyone that is in charge of training practitioners active in the field. For example, they relay the basic functioning, pros and cons of sanitation system and technologies for operation and maintenance purposes and/or urban wastewater management development.

Step 2: *Developing interview questions.* We define what to ask and construct a line of questions that help us extract the required information. We develop a persona template where we identify the main categories of required information and develop the questions based on these categories. The interview questions are aimed at projecting the interviewee into a typical work-setting. First, we ask about their general experiences, their function, attitudes and motivations as well as their organizational relationships and structure. Second, we elicit their specific pains and gains during an entire process-cycle. Here we focus in detail on the technology selection process and the use of existing products (tools and technologies). Third, we address mental processes by asking them how they would position them relative to similar actors in the field and ask them about their future expectations within the field. We have adapted the questions for three broad categories of practice: planning, research, and capacity development (Online Resource 1).

Step 3: *Contacting potential users.* We contact a number of actors that satisfy the definition of potential users and conduct a series of online, semi-structured interviews taking 40–60 min of each participants time. Furthermore, we rely on the “snowball principle” to find other potential useful actors to interview.

Step 4: *Analysis.* We transcribe and categorize the data in the predefined user groups. If answers are given from an inter- or transdisciplinary context, we will add those answers to the appropriate user category. For example, a planner can be affiliated with a university fulfilling a teaching position. This data will be categorized in the *planner* user group and answers corresponding to the teaching position will be used to consolidate the *teacher and trainer* user group.

Step 5: *Constructing personas.* We then synthesize a model of each user by matching the interview data to the corresponding categories in the format of a persona. These categories include the most prominent differentiator with other user groups, a picture of their background and position in the field, typical tasks, motivations, and attitudes towards the job and field, main challenges and gains, expectations for the tool and field, and the UX-design factors. Similarities in the responses are identified and help to highlight specific differences between the user categories. As an example, when two out of eight interviewees in the *planner* user group mention “case studies” as an important gain in their work we will indicate this as *case studies (2/8).* This allows us to order the importance of design criteria.

**Fig. 2:**
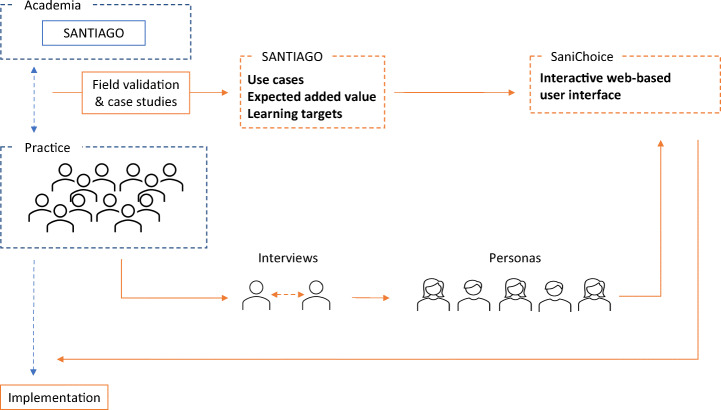
Developing an academic software, SANTIAGO, into a tool fit for practice, SaniChoice, by assessing results from field applications and interviews with international stakeholders from practice to construct personas through human-centered and user-experience design

Step 6: *Bridging research and practice.* We develop a framework where we couple our academic contribution to lessons learned from the practical application of SANTIAGO in order to create specific understanding and support for the development of SaniChoice for practice. Our methodology is summarized in Fig. 2.

## Results

### Advantages of SANTIAGO and Potential for Practice

SANTIAGO is designed for the following: (1) the generation of locally appropriate sanitation systems from a diverse and large set of technologies; and (2) the quantification of resource recovery potentials to support comparison of system options at the scale of an entire settlement. The aim is to enable a systematic consideration of technology innovations and sustainability criteria at an early stage of strategic sanitation planning. SANTIAGO is not intended to replace any existing planning frameworks that address the entire Structured Decision Making (SDM) process (e.g. CLUES^2^) but merely provides the tools to operationalize step 3 (identification of decision options) and step 4 (evaluation of options).

The two main advantages of SANTIAGO are: *(1) any (future) technology option can systematically be considered when generating sanitation systems*; and (2) *resource recovery potentials can automatically and ex-ante be quantified for a large and diverse set of systems by compiling international data and using uncertainty estimations.*

The example and practical applications of SANTIAGO showed that the model is capable of generating reasonable results. Specifically, it showed that there exists some key characteristics that influence resource recovery. These key characteristics allowed us to develop a number of recommendation for the development and selection of sanitation technologies and systems for resource recovery:

1. Prioritize short systems that close the loop at the lowest possible level (fewer treatment steps results in fewer losses);
2. Separate waste streams as much as possible. This does not necessarily lead to fewer treatment steps, still it allows for higher recovery potentials (e.g. through urine separation);
3. Use storage and treatment technologies that contain the products as much as possible and to avoid leaching technologies (e.g. single pits) and technologies with high risk of volatilization (e.g. drying beds);
4. Design sinks that optimise recovery and avoid disposal sinks;
5. Combine various reuse options for different side streams such as the reuse of urine and the production of biofuel from faeces.

The results also led to two key conclusions which will guide our future research. First, both the local appropriateness and resource recovery depend on technology interactions and system configurations and therefore has to be evaluated for entire systems. Second, there exist no unequivocal set of factors determining appropriateness of resource recovery. And, local appropriateness, resource recovery and other important sustainability indicators can be contradictory. This highlights the need for an automated software that is able to generate all valid sanitation systems and provides ex-ante quantification of their appropriateness and resource recovery potential.

Additionally, the applications showed that SANTIAGO brings several advantages over existing methods:

1. It is generic and therefore versatile to be applied to a diverse set of sanitation technologies and system options.
2. It is flexible enough to support the integration of novel technologies or decision criteria.
3. The systematic nature makes it reproducible and comprehensive: (i) the technology appropriateness assessment uses a set of clearly defined criteria and a standardized procedure for their quantification; (ii) an algorithm generates all valid sanitation system options; and (iii) it makes technical suggestions for each and every product and therefore enforces the consideration of entire sanitation systems.
4. A number of simplifications make SANTIAGO automated and it can therefore deal with a very large number of technologies and systems (over 40 technologies, and over 100.000 system options).
5. Uncertainties related to the technologies, their implementation, and the local context are explicitly considered. This makes the methods applicable at the structuring phase of decision making and enables an evaluation of the robustness of the results.
6. The SANTIAGO technology library compiles suitable input data for 41 technologies, 27 screening criteria, and transfer coefficients for four substances (phosphorous, nitrogen, total solids, water) based on international literature and expert knowledge.

These innovations lead to a number of advantages that can be achieved by the application of SANTIAGO in practice:

1. The set of decision options is diverse and thereby (i) opens up the option space with potentially more appropriate and sustainably options which one might not have thought of manually, and (ii) has the potential to reveal the majority of relevant trade-offs regarding the main decision objective, and thereby lowering the risk of impacting the final decision by this structured screening.
2. Because international performance data is matched with local information, more empirical decision making is enabled. This potentially enhances ownership and reproducibility.
3. The decision-making process is streamlined as the options are reduced to a manageable number of appropriate options only.
4. The option generation is based on decision objectives and is not limited to the knowledge and experiences of the involved experts.

The two main potential added values that we expect from the adoption of SANTIAGO in practice are (1) *to find appropriate sanitation systems which may not have been considered without using the software (algorithms + library). This helps to think out of the box, and possibly leapfrog to up-to-date knowledge.* (2) *To increase resource recovery and by that support a circular economy.*

These results from the field applications and case studies led us to define three possible *use cases* of a practical tool, the *expected added value* of its use, and specific *learning targets* that users should have understood after its usage.

### Use Cases

*Planning:* (i) Compare appropriateness of a set of technologies based on local conditions and appropriateness criteria; (ii) identify a set of locally appropriate sanitation system options*;* (iii) compare sanitation system options regarding different criteria including resource recovery potentials, complexity and costs level*;* and (iv) visualise trade-offs among different criteria and options, adjust options, select preferred options together with relevant stakeholders.

*Capacity Development:* (i) Overview on technology and system options; (ii) systematic appropriateness assessment for a given context (what to consider, and how to address); (iii) multi-criteria sustainability evaluation of planning options (what to consider, how); (iv) hardware selection as part of structured decision making for CWIS^3^ (SANTIAGO methodology); and (v) link to other tools and approaches for CWIS.

*Awareness Raising:* (i) Present comparisons to decision-makers to advocate for optimal system configurations regarding appropriateness and resource recovery; (ii) raise awareness about how much the system performance depends on the local context and the technology interaction (which cannot be evaluated on the technology level only); and (iii) present evidence about potential advantages of technology innovations.

### Expected Added Value

1. *Thinking out of the box:* enabling the consideration of a broad range of conventional as well as novel technologies and system configurations that one might not have thought of based on experience alone;
2. *A systemic approach:* enforcing the consideration of the entire sanitation value chain (from the user interface to reuse or disposal);
3. *Enhancing transparency:* providing a set of criteria to systematically assess technology appropriateness.
4. *More empirical:* matching technology data from international literature and experts with specific local conditions
5. *Multidimensionality:* using a multi-criteria decision approach to provide the user with an easy accessible overview on the performance of a diverse set of relevant sustainability criteria as a basis to negotiate trade-offs and find the most appropriate solutions for all stakeholders.

### Learning Targets

1. There exist many different technology options which can be combined into a large and diverse set of system options;
2. There are many different criteria for technology and system options, whose importance is dependent on the local context;
3. Criteria can be organised into ”fixed” for preselection (appropriateness) and ”to be negotiated” (involving trade-offs for facilitated multi-stakeholder final evaluation of sustainability);
4. Appropriateness depends on context, e.g. high water requirement is only a problem where water is not available);
5. Sustainability depends on preferences, e.g. stakeholders might disagree on the importance of costs over that of resource recovery;
6. Performance depends on technology interaction, therefore always has to be looked at for entire systems. For example, if one of the technologies is inappropriate, the entire system appears inappropriate, or if resources are already lost in an upstream technology, the potential of recovering the residual in a technology downstream is minimal.

### Design Requirements for SaniChoice

Our approach resulted in conducting 21 semi-structured interviews with international actors from the field of urban sanitation, active in development and developed contexts in the global North and South.

Their answers are combined into five distinct personas according to the pre-defined user groups. A full representation of the personas can be found in Online Resource 2. We learned that most of the actors work inter and trans-disciplinary. This notion has led to the cross-adoption of answers, i.e. answers from one category of users that correspond to another category are used to supplement other user groups. Specifically, we performed six interviews with *Capacity Builders*, incorporating the answers from one actor out of the *Planner* category; one interview with an E*ngineering Expert*, supplemented with the answers from two actors in the *Planner* category; five interviews with *Planners*, enriched with answers from one actor in the *Researcher* category; five interviews with *Researchers* complemented with the answers from one actor in the *Teacher* category; and lastly, three interviews with *Teachers and Trainers,* enhanced with the answers from three *Capacity Builders*.

We then extracted a number of features from the personas that we deem crucial for the design of a tool in order to be attractive for practice. First we identify main needs arising for all personas and second we extract the main information on experiences and feelings.

### Practitioners Needs

1. *Context Understanding*. Understanding is largely governed by case studies and consideration of political/organizational dimension. Most of the interviewees identify case studies as a crucial component lacking in many current approaches. Showcasing successes and failures (using tools such as SaniChoice) helps to make the translation from virtual to reality and to convince decision makers. Moreover, coupling case studies with contacts and a possible marketplace helps to match appropriate stakeholders for a given context.

(2) *(Bridging) Planning, Design, Decision, and Implementation.* Users identify interdisciplinary case studies and cooperation crucial to bridge the gap between planning and implementation. Specifically, linkage to (inter) national development plans, guidelines on understanding of both data-gaps and the level of centralization is key. Furthermore, it should be clear from the beginning how the tool fits into, and supports the entire process cycle. Especially, what does the tool offer for each stage of the process from design to implementation? The tool should be applicable to various contexts and scales. This includes urban and rural areas, as well as emergency, or school situations. The general public as well as experts should be able to work with the tool. On the one hand, the user should rapidly obtain an impression of, for example, the required details or feasibility of a certain sanitation system or technology, without being overloaded by the entire system complexity. On the other, the user should have the possibility for more extensive in-depth usage. For example, to plan and evaluate detailed sanitation systems or technologies, including all details needed to develop a sanitation system from design to implementation. At the same time, the tool should be easy to understand and its use mastered within a short time-span. This could be supported by clear guidance on how to interpret the results.

(3) *Access to Information*. There is a strong desire for a “one-stop-shop” platform that presents an overview of practices, case studies, publications, tools and approaches in the sanitation chain to ensure the user that they take into account all considerations needed. For example, there is a multitude of tools available that assist in the development of sanitation solutions. However, knowing when to use which or knowing what is available remains a challenge.

(4) *Additional Services.* Users require pre-packaged approaches, templates, and a platform where they can connect to stakeholders to help awareness raising. The platform should aid in levelling the background of the diverse set of actors in the field—having stakeholders speak the same language is one of the key-components that takes a good amount of time in current approaches. This specifically requires that (1) results should be available in a format that can directly be adopted in reports and presentations. This will motivate users to actually use the results were their respective stakeholders. (2) The use of language and appropriate visuals is crucial. Meaning can get lost in translation and inappropriate visuals could result in stakeholders to have prejudices against proposed solutions.

(5) *Quantitative and Qualitative Estimations.* Users want to obtain quantitative and qualitative performance data to narrow the scope of technology options and understand how they fit into an implementation context considering the enabling environment. When evaluating system or technology options, there should be a clear indication of trade-offs. Any use of data should be transparent and corresponding results not too complex in order to convince locals and decision makers of proposed solutions.

### Practitioner Experiences and Feelings

1. *Usability.* Users require explanatory videos and written guidance on the use and applicability of the tool. Differentiated complexity of use, but easy data input, visual output of the results as well as an understanding of how to interpret these results.

(2) *Accessibility.* Users require full functionality offline and on low bandwidth devices, accessible to everyone (not only experts) by differentiated level of complexity and an interactive design. The software should be open source.

(3) *Credibility.* Users define credibility mostly by the uptake of the tool in practice which is showcased by case studies and the backing from large organizations and peers. Evidence and uncertainty of performance data is important as well as full transparency in use of data, algorithms and continuous updated information.

(4) *Desirability.* Users require an overarching platform connecting different disciplines and creating understanding of the fields’ complexity by checklists and toolboxes that focus on the enabling environment. Further, it should host ready to use materials to be adopted in presentations, reports, videos and posters.

Next, we develop a generic framework (Table 1) in which both aims of our research can be coupled, and clear implications for the design of SaniChoice arise. A full representation of the framework can be found in Online Resource 3.

**Table 1:** A framework indicating specific understanding of each user category on the basis of personas including main needs and expectations, linkage with requirements arising from practical applications of SANTIAGO, and resulting concrete desires from practice that can be translated into design specifications of SaniChoice

**User understanding extracted from personas (a)**							**Requirements extracted from SANTIAGO field-testing (b)**			**SaniChoice** **design and architecture**	
		**Capacity builders**	**Engineering experts**	**Planners**	**Researchers**	**Trainers and teachers**	**Use cases** (1 - 3)	**Expected added value** (1 - 5)	**Learning targets** (1 - 6)	**Synthesis (a & b)**	**SaniChoice provides**
**Practitioners (P)**	Tasks										
	Thinking and attitudes										
	Motivations										
**P Needs**	Context understanding										
	(bridging) planning, design decision, and implementation										
	Access to information										
	Additional services										
	Quantitative (1) and qualitative (2) estimations										
**P Experiences**	Usability										
	Accessibility										
	Credibility										
	Desirability										

The combined understanding from field applications and interviews are being developed into an easy-accessible web-version of SaniChoice. Specifically, it targets the differentiated backgrounds of potential users and provides linkages with platforms such as the *Sustainable Sanitation Alliance* (SuSanA) library. It integrates an online *Compendium of Sanitation Systems and Technologies* with SANTIAGO and makes available training packages that can be used to (1) understand the use of the tool by descriptions, videos, and expert functionalities; (2) train future users; and (3) provide a step-by-step guidebook of how to integrate SaniChoice in the planning process. A tentative architecture of the tool is presented in Online Resource 4.

## Discussion

Our methods are based on a synthesis of earlier research applied to possible use of SANTIAGO for practice and the specific extraction of user requirements on the basis of personas. Pre-defining user categories might lead to potentially missing the full scope of future users. For example, we have not considered those active in implementation and operation of sanitation technologies. Furthermore, using personas as our main tool to extract user requirements from practice is one of many different approaches available in human-centred design [37]. The choice of personas is mainly guided by prior experience. Moreover, there exists a large body of research behind the design, analysis and implementation of personas in developing user-products. An in-depth analysis of potential pitfalls would contribute to further detailing the personas to our needs and likely further refining specific user requirements.

The interviews are conducted with only a selective set of actors resulting in possibly biasing our results. First many have an engineering background and are active in or close to an academic research setting, and second, all interviewees are active in coordinating and supporting roles. Having no interview responses from the implementation level, e.g. construction workers, and those in charge of operation and maintenance of systems possibly leads to the tool being designed for coordinating efforts and could result in further increase of the implementation gap instead of overcoming it. On another note, further refinement per region would contribute to the likelihood of the tool being adopted, because now we consider a broad scope of regions and settings in which respondents are active.

The validity of responses to interview questions could be flawed for a number of reasons. First, the level of question detail could not be answered by all interviewees, hence the validity of some answers are flawed as a result of a small sample size. For example, the questions about selection of decision options were too specific for most of the interviewees. Second, the semi-structured nature of the interview results in different story development and thus responses. Retrospective linking of answers to corresponding questions might not reflect the exact attitude from a respondent. Third, some interviewees read through the interview questions prior, whereas others did not possibly resulting in different level of understanding and answer detail. Fourth, time restrictions limited the scope of questions that could be treated.

Analysing the data was complicated because most actors are active in inter- and transdisciplinary functions. For example, the political dimension of sanitation solutions is more clearly mentioned as a crucial factor by capacity developers than planners. And, this resulted in difficulties in both fitting the actors into the pre-defined user categories, and the cross-adoption of answers to appropriate user categories.

The results suggest that the integration of SANTIAGO results in clear benefits for practice. The validity could be further improved by addressing the aforementioned discussion points and by showcasing the use of SANTIAGO in additional application examples. Additionally, we deem SaniChoice valuable for policy makers in developing measures that effectively target sustainable development goals 6 (clean water and sanitation), and 11 (sustainable cities and communities), especially because uncertainties are considered that allow for e.g. scenario building.

Last, the genericity of our approach is clear from the methods as well as the resulting framework. This specific roadmap can be applied to any academic software in order to make it available to practice. However, we are not able to answer to the full scope of user requirements that arise form practice with the development of SaniChoice alone. This would need a larger scope of platform in which SaniChoice is linked to other tools to provide guidance along the entire sanitation chain.

### Conclusion

This research showed that the practical application of SANTIAGO results in clear requirements. Primarily, local appropriateness and resource recovery potential strongly depend on technology interactions and thus has to be evaluated for entire systems. This highlights the need for an automated approach as provided by SANTIAGO. Moreover SANTIAGO can bring a number of advantages over existing approaches: (i) it is systematic and reproducible; (ii) it opens up the decision space with novel and potentially more appropriate solutions; (iii) it makes international data accessible for more empirical decision making; (iv) it enables decisions based on strategic objectives in line with the sustainable development goals; and (v) it allows to prioritise appropriate and resource efficient systems right from the beginning.

Developing the personas shows that we arrive at meaningful design requirements that would enable the uptake of SANTIAGO through the SaniChoice tool in practice. In the main, the users require transparent context understanding to help bridge the gap from planning to implementation of sanitation solutions. This is mostly achieved through the presentation of case-studies (that use tools such as SaniChoice), alignment with (inter) national development plans, differentiated complexity of tool-use (general public and experts), and user guidelines. These main requirements should help the user in developing appropriate and sustainable services for their specific roles within the sanitation chain. With these recommendations, we hope to be able to develop a tool that enables practitioners to systematically consider novel technologies in planning entire sanitation systems. Specifically, to prioritise locally appropriate, more resource-efficient sanitation system options at an early planning phase thereby contributing to circular economy and sustainable development.

Future research could draw on the need for an overarching platform that provides a one-stop shop to guide practitioners through the entire process cycle. A first step towards this platform is presenting SaniChoice to practice and iteratively develop the tool into something covering a larger extent of the process cycle by close collaboration with the users and existing tools and platforms.

## Supplementary Information

ESM 1 (DOCX 25.4 kb)

ESM 2 (DOCX 81.3 kb)

ESM 3 (DOCX 31.1 kb)

ESM 4 (DOCX 500 kb)

## Data Availability

Performance data of practical applications of SANTIAGO is available upon request as well as the anonymized transcripts and interview recordings upon consent of the interviewees.

## Footnotes

1 Safely managed: “Use of improved facilities which are not shared with other households and where excreta are safely disposed in situ or transported and treated off-site” https://washdata.org/monitoring/sanitation [Accessed on 01.02.2021]

2 Community-Led Urban Environmental Sanitation Planning. Available at: www.sandec.ch/clues

3 Citywide Inclusive Sanitation Planning. Available at: www.sandec.ch/cwis
