# Supplementary material for: Closing Water and Nutrient Cycles in Urban Wastewater Management: How to Make an Academic Software Available to General Practice
Source: Circ Econ Sustain. 2021 Jul 15;1(3):1087–105. doi: 10.1007/s43615-021-00073-6 (PMC8679645; doi:10.1007/s43615-021-00073-6)
Supplement: Supplementary file 1 — (DOCX 25.4 kb) [file 43615_2021_73_MOESM1_ESM.docx]

Closing water and nutrient cycles in urban wastewater management: how to make an academic software available to general practice

Johann S. Schuur^a,b^ and Dorothee Spuhler^a^

(0000-0002-0924-2319, 0000-0002-1379-6146)

Corresponding author: Johann S. Schuur, [jschuur@ethz.ch](mailto:jschuur@ethz.ch)

*^a)^ Eawag, Swiss Federal Institute of Aquatic Science and Technology, 8600 Dübendorf, Switzerland.*

*^b)^ ETH, Swiss Federal Institute of Technology, Institute of Science, Technology and Policy, 8092 Zürich, Switzerland.*

Description of content: This file contains the semi-structured interview protocol that was used to develop the personas

**Interview protocol**

We merged three different interview protocols into one. Differences are small, yet tailored to three broad areas of working backgrounds from the interviewees – planners, researchers, and trainers or capacity builders - and emphasized in italics.

*OVERVIEw*

- What is your expertise in urban sanitation?
- How do you apply this in your current job?
- How would you describe your function in urban sanitation?

#### ATTITUDES AND MOTIVATIONS

- What is your motivation for your job?
- What do you need to *be successful* *in urban sanitation planning/researcher of sanitation systems and technology options/develop successful trainings or capacity building in urban sanitation*?

*RELATIONSHIPS AND ORGANIZATIONAL STRUCTURE*

- With whom and how do you normally interact in *planning for urban sanitation / researching urban sanitation / such activities*?

*PAINS AND GAINS FOR OPTION SELECTION AND EVALUATION (PROCESSES)*

- Can you summarize the steps in a typical *sanitation planning / research / training or capacity building* process?
- Did it change over time? If yes, how?
- What are factors that facilitate your work?
- What are the challenges that you face?
- How would you identify *decision options* *in a participatory decision-making procedure (such as CLUES or Sanitation 21) / decision options that you would further investigate / sanitation systems or technology options in a training module*?
- Where would you start and what would you do next (how would you proceed)?
- When would it be finished?
- What criteria do you use?
- What selection procedure do you use?
- How long does this typically take?
- What is your main challenge when coming up with *decision options* / options for sanitation systems or technologies?
- What do you think would help you to do it better?
- How would you compare and evaluate decision options for sanitation systems/technologies?
- Where would you start and what would you do next (how would you proceed)?
- When would it be finished?
- Are there any main characteristics on which a comparison is made?
- What is the most difficult trade-off to make?
- What is your main challenge when comparing options?
- What do you think would help you to do it better?

#### TOOLS AND TECHNOLOGY (PRODUCTS)

- What are the tools you use to accomplish your tasks or goals (e.g. frameworks, software, literature, consultant)?
- When would you use a web-based platform instead of relying on your own skills/experience/research?
  - In case you wouldn´t, what is the main reason?
- Do you feel that you are aware of all the tools/platforms available?
  - If yes, how do you keep yourself informed?
  - If no, what could we do to keep you “in the loop”?
- What traditional (i.e. analogue) tools do you use to accomplish tasks in your job?
- What digital tools do you use to accomplish tasks in your job?
- Do you like to use a tool why or why not?
- What do you value most from using existing
  - Principles (general theorem accepted by international community providing basic guidance, e.g. SDG 6)
  - Approaches (framework or methodology aimed at putting principles into action, e.g. CLUES)
  - Tools (instrument supporting operationalization, e.g. SANTIAGO)
- Where do any of your tools lack usefulness?
- When we come up with a web-based tool, on what type of device would you use it mainly?
- In your capacity, what would be the most helpful functionality of the platform (e.g. learning, training, teaching, planning)?
- How would you like to access this functionality on a website?
- How would it look like and what are the steps it would allow you to do?

#### MENTAL MODELS

- What do you wish you had that would level you with, or give you an advantage over similar actors involved in urban sanitation *planning / research / training or capacity building*?

#### PROJECTING INTO THE FUTURE

- If we would get to talk in about 5 years’ time, what would be different in the *field of urban sanitation planning / research field of urban sanitation / field of urban sanitation training or capacity building*?
- What would be different in *planning / these* processes, and the selection of technology options specifically?

#### WRAPPING UP

- Is there anything that I haven’t considered and you find crucial for me to know?
- Are there any questions from your side?

#### General PAINS AND GAINS

- In general, in your work, what is the biggest challenge you face? Why?
- After a typical workday, what is usually still on your mind?
- What would make your work easier?
