# Supplementary material for: Closing Water and Nutrient Cycles in Urban Wastewater Management: How to Make an Academic Software Available to General Practice
Source: Circ Econ Sustain. 2021 Jul 15;1(3):1087–105. doi: 10.1007/s43615-021-00073-6 (PMC8679645; doi:10.1007/s43615-021-00073-6)
Supplement: Supplementary file 2 — (DOCX 81.3 kb) [file 43615_2021_73_MOESM2_ESM.docx]

Closing water and nutrient cycles in urban wastewater management: how to make an academic software available to general practice

Johann S. Schuur^a,b^ and Dorothee Spuhler^a^

(0000-0002-0924-2319, 0000-0002-1379-6146)

Corresponding author: Johann S. Schuur, [jschuur@ethz.ch](mailto:jschuur@ethz.ch)

*^a)^ Eawag, Swiss Federal Institute of Aquatic Science and Technology, 8600 Dübendorf, Switzerland.*

*^b)^ ETH, Swiss Federal Institute of Technology, Institute of Science, Technology and Policy, 8092 Zürich, Switzerland.*

Description of content: This file contains the five different personas that were developed upon analysing the interview responses.

Capacity builder – “Chloe Chass-Dufour”

The following is based on **9 interviews** with capacity builders in the field of urban sanitation. We define a capacity builder as a person working in a capacity building or development agency as well as in institutes and foundations that are involved in the development or backing of sanitary solutions for urban settlements (in developing countries).

For those interviewees that have given similar answers, we have added (n/m), indicating the stringency of an aspect. For example, when two interviewees indicate “local stakeholders” as being important it would look like: *local stakeholders (2/m).*

| General  Chloe Chass-Dufour | | | Photo |
| --- | --- | --- | --- |
| A quick take on  Chloe is a 52 year old capacity developer with a background in environmental engineering. After her studies she worked on developing action plans for the provision of basic urban services in developing context mainly in the Global South. She worked 12 years on urban sanitation projects in India, Thailand, Ecuador, and Peru. She worked shortly for Helvetas where she focused on small-scale projects in India. In South America she focused on drinking water and sanitation. Moreover, she was shortly involved in drinking water treatment and river management projects in Germany as well as China. She founded the Environment and Public Health Organization (ENPHO), which she led for 15 years.  The most enjoyable part of her job is the interaction with stakeholders and connecting them with experts to find appropriate solutions that fit in a local, often resource poor, setting.  At the moment, she is the Deputy Director of Eawag, an institute focused on aquatic research, based in Zwitserland. | | | |
| Goals and customer jobs  Her duties include guidance and development of project proposals and developing mission statements. She delivers advisory services for large donor organizations and development banks such as SDC and SECO in Zwitserland, and the Asian Development Bank, the World Health Organization, and the World Bank. Her expertise is also requested for NGOs like Oxfam and Helvetas.  On a different scale she is in touch with national government agencies, engineering departments, universities, other capacity builders and foundations, and occasionally with local level municipality actors through partner organizations.  Usually, her role stops before the implementation phase, but because of her background she is often consulted for the monitoring of cases. Specifically, she assesses which tools are more relevant, if developed tools are used and used properly by e.g. checking if they are marketed by partner organizations. The main indicator is that they should be used by the intended target audience in the field. Recently she was involved in the monitoring of a tool that is assessing WASH infrastructure in health-care and schools. It is used by Terre des Hommes and is now also the basis for Swiss Water and Sanitation Consortium.  She sees herself mainly as a facilitator, because she is continuously occupied with stakeholder engagement, understanding the disconnect between supply and demand among them, and identifying barriers and constraints that arise in the field. She brings stakeholders together by showing the broader concepts of the City Wide Inclusive Sanitation (CWIS) approach, indicating which improved sanitation solutions could be achieved, showing new ways of thinking and possibilities for data collection (methods and tools) and a shift to urban sanitation schemes.  When a new project commences, she first identifies the options by creating ***understanding of the governmental plans and budgets (2/9)***, then the local needs and gaps including environmental considerations, and researches existing training or capacity building programs. Then the capacity building process starts by  customizing this knowledge to approaches fit for the local context. This customization is greatly benefitted by the creation of small enterprises that she sees as pilot projects which are usually the joint effort of NGOs, policy makers, and local stakeholders. Thorough assessments can take up to a year. | | | |
| Motivations  Although it is time consuming, she enjoys to ***bring all backgrounds of stakeholders on the same level (2/9)*** by creating trust. This makes the work fun and very interesting. She sees that many ***people that do not understand the issue (2/9),*** which motivates her to show all the possibilities in the sanitation chain.  Her work in sanitation is driven by ***witnessing the striking situation (2/9)*** of open defecation in Bangladesh. Sanitation is the biggest health crisis in many countries in which she worked.  She finds urban sanitation capacity building critical, because she sees a big capacity gap, but feels that the field is now starting to make forward momentum. | Attitudes  She envisions the development of ***soft-skills (2/9)***, social engineering, and stakeholder engagement over industrialized technological approach. ***A large part of the solutions are often not only technical (4/9)***, i.e. the entire ecosystem needs to evolve***.*** Therefore the choice of appropriate partners (are they up for the task?, stable?) in an interdisciplinary team is a large and very important part of the work albeit time consuming. She always puts the context first, because community-based projects clearly perform better than four decades of top-down decision making. Especially because they are the ***key stakeholders that can influence*** (block or support) ***the process*** ***(3/9)***.  Being supportive is identifying and addressing different layers of the real actors (those with deep understanding of practical or theoretical side of things) before developing anything. It is important to find a genuine niche to come up with solutions for stakeholders that answer to what they are actually looking for. They need to be convinced. One needs to understand the best place to market and reach larger audience, but the real actors (e.g. users, informal sector) do not care about advertisements ***involving them in the platform is therefore crucial (2/9)***, this can be done by creating local market for services, or formalize their services by private partnering with public. Further, costs are sensitive thus people should understand the extra costs that they will need to endure.  Not much will change fast as monitoring is often not part of the planning process, but it is desperately needed. However, she expects every International Foundation Institute to have unconventional solutions in portfolio in about 5 years time and believes that investments will be increasingly nationally and regionally led. Therefore it is important that ***tools are utilized (2/9)*** and the platform’s reflection of international norms is ***linked with regional and nationally based directives*** (norms and standards) ***or projects (2/9)*** in order not to set up separate efforts, and allowing for proper assessment. It is needed to confront outdated and non-sustainable stringent norms and standards. Second, they should not only be directed to INGOs and Development organizations, because then they might not be very helpful, i.e. they should be targeted.  She expects that capacity building programs such as workshops, trainings, conferences and seminars, courseras and MOOCs, and monitoring will be ***mostly online (4/9)*** in 10-20 years. Even virtual reality for meetings could be a serious option. If the platform can provide this, organizations such as Helvetas are not needed anymore and can evolve in something else. The increasing demand for capacity building should be utilized and makes her hopeful since it spreads the many innovations and understanding from the field. However, she is skeptical about replacing personal context, and the level of exchange on online platforms. Afraid consumers lose their ability to place short contents into bigger context.  -  Likes to combine bits and pieces from different frameworks, methods and tools.  -  More interventions on sanitation.  -  Decision makers should be guided and this is not happening. Show range of possibilities and provide all information (pros and cons), ensuring long-term planning including needs for operation of system, design, and the ***life-cycle costs (2/9)***.  -  Sees sanitation as one of services next to other urban services and infrastructures.  -  Everyone is working on smart-board for sanitation design.  Tool can be helpful to establish common denominator about process and steps.  Where does platform stop and expert take over? Should the systems take a life on their own, or should they keep being reliant on platform?  Processes should be made visual.  Technical details should not be responsibility of platform, but make visible what is connected to what, where does data go.  -  Who should be in charge, who should manage. | | |
| Pains  Administration  ***Paradigm change*** with authorities ***is most difficult (2/9).*** Language of different stakeholders, and getting everyone to speak the same language. In addition, ***get people*** with other priorities ***motivated to identify themselves with situation (2/9)*** (which is a world-wide problem). Which is exemplified by a fundamental lack of understanding of water cycle and proposed solutions.  ***Time to look for the right tool (2/9) and resources (2/9).*** Very contextualized tools require a lot of time and (prior) knowledge  Respect of experts towards rural people.  Planning based on shaky data/samples taken some years ago.  -  Most tools not accessible/visible on mobile phones.  -  Rapid urban and unplanned growth. Problems with other services already available.  -  Difficult to go in depth and get a good grasp of situation as an outsider. This is further hampered by the fact that it is really hard to replace real-live discussions, even with zoom.  -  Options are in limited number, not right scale, or only existing technologies are considered.  Understanding what is available in the market, what is functioning, what is problem, what to do to manage problem. Who is provider, what are costs, who should be responsible, who does maintenance.  Focus on decisions that are community-led (only works in rural context), in general community does not understand and do not have the time.  Missing who is the target audience for tools, and respective customization.  Integration of tools in formal programs is not done properly.  -  Changes in local leadership.  -  Striking balance between platform that is not too hard to deal with nor to maintain. Trade-off between level of sophistication and ease of use.  - | | Gains  ***Partnering (2/9)*** with a ***good and motivated team (2/9)*** of ***people from different (technical) fields (3/9)*** to find solutions and knowing that they can stick to the process over time. Work with locals as much as possible. Get people to respect each other by ***clear communication*** **(2/9)**, clearly assigned roles and responsibilities, clear guidance, good ***working environment*** ***(2/9)***, and understanding of the (size of the) problem. Important is to not assume that you have the answer.  WHO water safety plans, rural drinking water framework.  SuSana website (especially “word search”, and “search by categories”, “search by country”), ***FSM toolbox (2/9)*** (further reading is weakest), ***Compendium (6/9)*** to check information for the community of practice.  WOCAT database (University of Bern). ***CSD (2/8)***, Shitflow diagram, FSD to structure and communicate. Guidelines from ***Sanitation 21 (2/9)***, ***CLUES (4/9)*** (but less on community led), sanitation service chain for FSM, City Wide Inclusive Sanitation approach. Indian capacity building platform NIUA (which is supported by 6/7 large capacity building organizations). Surveys to evaluate WASH in schools and health-care facilities, e.g FACET, and the BLUE-schools approach which helps to understand process.  Further gains are found in presenting and public speaking skills. Online questionnaires and courses, videos, grey-literature and documentation of ***case studies (2/9)*** as well as novel technologies.  Field-trips can be an effective method to ***change mindsets (2/9)***. And in combination with the correct guidance helps to obtain ***resource allocation from governments (4/9)***. Regulations activities and national development plans needed to plan urban development should move in more or less same time-scale. Here, consultancy and continuous advocacy for e.g. national sanitation plans and joint-force conventions by better coordination to convince governments, talk to policy makers and donors to ensure endorsement and commitment from top-level. This makes it easier to reach institutional change.  However, it needs to be clear what the option space for local, political involvement, distribution of managing factors and setting up of new system is. Better coordination is very important to reach institutional change.  Exposure of community leaders and going through collective “piece de connaissance”/collective awareness. | Expectations  “Must-haves”  Mastering of tool within half a day, by itself without training.  Convincing case (policy briefs, video courses, conferences)  Up to date information from WHO, FAO. International guidelines.  Information on functioning and applicability of technologies.  Examples of ***case studies (2/9)***, positive, long-term, failures/challenges.  Concrete outcomes and the consideration of ***enabling environment (2/9)***, as well as distinguishing between different spatial levels.  Small explainer videos indicating applicability to municipal utility, or more general to consultant. Making it directly apparent that it is effective, ***less time consuming (3/9)***, comprehensive, and has an advantage as compared to existing platforms. Data collection should not be too difficult.  Readiness of capacity packages, a library and how to connect to organizations throughout the entire chain.  Simplicity and a limited number of steps.  Powerful ***visualizations (2/9)*** such as the Shit Flow Diagram.  Should help to build common denominator.  Main platform functionality  Create a ***one-stop shop (2/9)*** to have a full overview of what is required by a  ***database/knowledge hub*** ***(2/9)*** including ***existing tools (2/9)*** needed to collect data, and conceptual overview of different approaches to ***understand context (2/9).*** Resource ***planning (3/8)*** process, complete process cycle learning capacity on platform itself.  Review of existing systems, ***actual interventions/ implementation (2/9)*** and timeframes as well as action plans and support to management and operation (including monitoring and evaluation). I.e. it should allow for a ***complete process cycle (2/9)***. |
| User experience  **Usefulness** – Should be able to be shared with colleagues. Timeframes of implementation and planning process. Planning parameters (how to plan system, guidelines to look at for given country.)  -  These are some options to consider further, with some ***features highlighted that might have to be looked into (2/9)***  Platform that narrows down option space is very helpful.  -  Should be clear how capacity building fits into process.  Instantly see visual output.  -  Being useful without being a blackbox.  Top-notch figures (SFD are good outputs) to be directly adopted in report, very important for effective communication.  -  Video, presentations and process explanations.  Operationalization/ getting Compendium more in flow.  Visual aspect of seeing effects of outcomes.  Import data from mobile survey, auto-factoring in other design variables such as environmental factors, transport, distances.  -  Appropriate to context of users, e.g. slick for the minister of this and that country versus simple and effective for local practitioners.  -  Provide specific evidence about projects making a difference in a community, showing benefits with testimony from e.g. government shows that we are legitimate and that we have value to offer.  Integrate how the designs fit into different local norms and standards.  **Usability** – Introduction ***videos (4/8)*** to download and stream (why tool, what does it do, and how to use it, how to engage and how to judge outputs) ***Roadmap/Written guidance (2/8)*** to print and take explaining tech, how to use the tool (max 10 pages): what can it do, who is it for, outcome look’s like this. This is how to use it.  -  Not too many different formats..  -  Easy to learn.  -  Small survey eliciting what someone is looking for and suggesting relevant information.  -  Series of tutorial videos describing what is happening in each step and what platform can be used for.  **Findability** – Sharing feature, links to include in whatsapp.  -  get quickly to content that is relevant  **Credibility** – Should be reliable. Good manual concisely describing technology, should not highlight technology that is favourite of institution.  Provide suggestions along with a warning: “based on your input the output is highly uncertain, if you would do this and this we can provide a more accurate solution”. Uncertainty range.  -  Likes design of Mock-up: here are options, these are the tradeoffs.  Provide benchmark of ***where tool has been used in case-studies (2/8)*** by e.g. reports to see how well it works in practice.  Integration of existing tools  -  Feedback from colleagues, knowing how it works and limitations. Are other tools or steps needed after? Partial output makes people question validity.  -  Should be used everytime capacity building is done.  -  Funding by international donor foundations.  Technical, but possibility to share information in simple way to population to explain why decisions need to be taken, especially when no consent is given allowing for transparency and participation.  Stakeholders willing that their data (of water flows) is with Eawag, in the cloud, shared with others?  **Desirability** – Click system together, having SANTIAGO compute appropriateness score of that compared to what system SANTIAGO comes up by itself. Database that is updated once a year. Interactive video or poster maker: toolbox from which e.g. non-sewered or sewered options can be compiled into poster.  Feature dealing with 1) ***data-gap (2/8)***, 2) basis knowledge about case/watershed by e.g. suggesting appropriate tools.  Projection of planning stage, incorporating future developments in e.g. energy sector, helping investment decisions.  -  Checklist for enabling environment (addressing key-requirements of each technology).  -  If platform provides insight in other countries water flows, it will attract a lot of users.  Worldmap with information. Add-like proposing of options similar to what user is going into.  This is subsidy from government, this is needed from the user (these are benefits and this is what it’s costing).  Section with time-stamps/markers showing what happened X many months after project, which can be filled-in by users.  Global access platform where all sanitation systems are described in good detail, which can be made country specific. Indicating these are agreed technologies, these are used, this is how the function, this is process.  Big data on benefits, how many safely managed sanitation through use of platform.  **Accessibility** –  *-*  ***PC in office (2/8)*** for management and administration, ***and mobile devices (3/8)*** for top-level planners and policy makers. It should be free (open source is caveat in successful platforms), web-based and offline version (available as download to start remotely in the field).  Take users through scenarios of different measures to be taken by making it like a game/***interactive (3/9)*** helps to connect to the right people and doesn’t feel like it is hard work nor that it can only be used by experts.  ***Accessible by*** ***any*** ***mobile data device (3/9)*** and ***Apps (2/8)*** with limited bandwidth  Visuals should also speak to local population and the choice of language is very important, subtitles for videos. Even though English is second language, it is not always spoken even in governmental positions. Open. Get it out of realm of conference stages and bring it down to those clicking the buttons.  Some say CLUES is too complex! | | | |

Engineering expert – “Renjal Depali”

The following is based on interviews with **three engineering experts** in the field of urban sanitation. We define an expert as a wastewater/sanitation system expert working in R&D or a consultancy firm, that is employed by any institution to analyze, develop, and give advice on wastewater technologies in urban settlements. Specifically to support urban sanitation planning.

For those interviewees that have given similar answers, we have added (n/m), indicating the stringency of an aspect. For example, when two interviewees indicate “local stakeholders” as being important it would look like: *local stakeholders (2/m).*

| General  **Renjal Depali**  Tackles urban sanitation design problems with his local circle of engineers.  “In general I do not like to use tools, because they take time and the usefulness of the outcome is unsure” | | | Photo |
| --- | --- | --- | --- |
| A quick take on  Renjal Depali is a 37 year old, Nepalese engineering consultant with a background in civil engineering. He closely worked with Eawag after which he continued his commitment to the design of sanitation technologies as part of ENPHO. He is the lead-designer and works on a subset of the urban sanitation planning process. He enjoys the intimacy of a small team and rather trusts on the expertise of his peers and the validity of physical case studies, as opposed to abstract designs, tools and frameworks. He therefore likes to be in the field, and enjoys the social aspect of problem solving. His primary function is designer and is slowly transitioning towards the planning of faecel sludge management (FSM) systems. The reason for this is that he now sees the design as a subset of the larger thing: planning. | | | |
| Goals and customer jobs  Renjali is responsible for the design of treatment plants that treat faecal sludge, and is partly involved in planning of FSM Specifically, the development of a business model for FSM. His main goal is to reduce open defecation and to inform people of the importance of considering quality over quantity considerations when it comes to urban sanitation. He hopes that in five years time the paradigm has changed from sewer-based solutions to decentralized, natural-based solutions. Here he thinks that the adoption of tools can form an instructive role, albeit simple and understandable by the people that actually use the systems and technologies.  Designs are developed in the office, where he works on a laptop. He interacts on a community-level.  At the moment he is also involved in a project “The end of waste water”. He helps develop a living database (one that is updated), and incorporates data mining to assess the performance of technologies in terms of resource recovery from grey literature, journals, etc.  Addressing a new problem, he first analyzes the current situation including existing technologies and practices, which is followed by a cost consideration. This mainly results in a reduced set of technologies that can be further investigated, because he is restricted by funding and guidelines from e.g. the Bill and Melinda Gates Foundation and GIZ. His designs need to be simple, smart, and user friendly. When delivering a system design it is time to connect to e.g. government officials (the ones that he is supposed to answer to), and many of these are social scientists. | | | |
| Motivations  Renjali is interested in the technical side of the sector. After understanding the importance of treatment plants in Nepal he saw that it was a good time to get into the field after his studies. At the moment sanitation is still more of a formality in that part of the world. He finds it a very interesting field to work in, because the problems faced are subject to factors such as political and legal structures, behaviour, technological ability, and time constraints. He likes puzzles, specifically the design of treatment plants that are as small as possible, and to help people where he learns how to weave his engineering back-ground into a more soft-skill approach. He mainly focusses on resource recovery, because a shift is needed to a more circular economy. Considering the environmental aspects allows him to be part of a paradigm shift to mixed service delivery and aid the development of a more sustainable approach. | Attitudes  He believes that success is dependent on having a network of young and highly motivated people that share knowledge and ideas. It is important to integrate all aspects when designing a system, and in the main he finds that success can be pinned down to aligning the asymmetry of knowledge. This includes the presentation of good case studies and technologies. Access to information, and the understanding of the context is thus very important. Specifically, knowing the most important system criteria.  Consultants are expensive putting another constraint on time, and therefore requires fast decisions to be made.  He bases himself as much as possible on natural forms of treatment, because mechanized systems are difficult to operate and maintain. However, his experience with popular systems and availability of construction materials often determine the suitability of a system.  ***He doesn’t use tools that much (2/3)***, but has heard about the FSM-tool a lot in the FSM world. The main reason for not using the tool is because he hasn’t had the chance to use them. Slowly he starts to see their benefit, but isn’t completely convinced yet. He likes it better to go into the field and design stuff with the team, contrary to NGO’s that do need to use tools as mandated by their clients. He believes that more experience, access to and interaction with people that are at frontiers of the field are more helpful.  In general, he expects that the approach to sanitation won’t change a lot, but people might start to understand importance better. | | |
| Pains  The communities that he works are mainly interested in the future of their kids, which involves education, good homes and good food. Sanitation is very low on the ladder. In addition, ***people do not understand the different nature of Faecel Sludge Management (FSM)*** ***(2/3)*** and that there is no one-size fits all solution, such as believing that waste water treatment is enough to also handle the shockloads of FS. People do not think that all these quality and quantity consideration are necessary, only if they see the value of selling recovered resources (even if these are not financially viable!).  ***Costs for novel technologies and available finances (3/3)*** are hard to estimate.  In many cases, novel technologies are too technical to be seriously considered because local capacity cannot ***ensure these systems to continuously operate (2/3)***. Moreover, replacement of parts of mechanized systems is hard because they need to be imported and this takes time.  In developing solutions he is challenged by ***limited time (2/3)*** and time horizon (designs are typically done for five years, because of a high learning rate), lack of creativity, a lock-in of old technologies and satisfying the client. The latter is challenging because he is not being trained in providing proof and evidence for calculations which results in many systems to be overdesigned.  Platforms that should help in this process are a pain, because they generally contain ***too much reading material (2/3)*** and the learning curve to the use of new tools is too high to seriously consider. This includes analog tools such as the Compendium for Sanitation Systems and Technologies. It would be helpful if this could be made interactive, but solely dragging around things as in the online compendium is not very useful. A system builder is the main thing that is missing, because many options are neglected in the design process. And he struggles when household surveys haven’t been done (properly).  Further, the internet connection in Nepal is often flaky, and in the field he would not be able to us internet at all. | | Gains  Education, and having a background in engineering, frameworks providing clear steps to follow, and having experience with similar cases help him to develop sketches and quantity estimations. Here, more monitoring and ***data collection*** ***(2/3)*** of existing technologies would help him to size and develop better systems. This includes the sampling of quantity and quality of sanitary waste, which is aided by a good familiarity with excel and the use of the Shit Flow Diagram (SFD).  Furthermore, the understanding of the local situation is enhanced by random sampling methods and household surveys about behaviours.  The dialogue with his network, i.e. peer-to-peer information-sharing to tap into the experience and suggestions of others is very valuable. The growth of the understanding in FSM is an outcome of this. This is enhanced by making presentations or animations. | Expectations  “Must-haves”  A must for the website is the ease of access to what is relevant for him. This is case-study information, combined with short video clips, and access to peers to share knowledge about certain projects. It needs to be as simple as possible and should include sludge quantity estimation and the visualizing of treatment processes.  Main platform functionality  His main use of the platform would be the technology applicability filter and planning. It should be detailed enough to carry out a sustainability assessment on the basis of the results. From this he wants to be able to start designing for the system or situation at hand. |
| User experience  **Usefulness** – He wants to have a solutions that doesn’t require other tools or frameworks to be able to implement the outcome into practice. Because he is not so good in the presentation of results, he want the tool to present him with graphs, outcomes, and facts that can be integrated into his reports directly. Furthermore, he finds it crucial that the results provide practical suggestions as to the dimensions of a technology, what materials it uses, and where he could find contractors that would be able to build the system.  **Usability** – ***Explanatory videos (2/3)*** (max. 7 min.) that cover the whole scope of sanitation will help him determine if the tool is useful for him or not. He likes the FSM toolbox, but doesn’t like to have to log-in and wait for approval. He does not want to go through complicated registration processes, but likes to have a quick and clean overview of the why, what, and how of a technology. It would be helpful if it would run on a phone.  **Findability** – He directly wants to access the specifics of sanitation technologies.  **Credibility** – He want to know what is new in the field, and he wants to know about previous projects where these technologies are implemented. Renjali will use a tool or platform once he ***knows what the outcome is (2/3)***, it is ***used (2/3)*** and talked about by his peers (like the FSM toolbox), it works and saves time, and he knows ***how to interpret the outcome*** ***(2/3)*** or what can be done with it. It needs to be widely accepted and supported by large organizations such as the WHO, BGF and GIZ. It needs to be able to explain things scientifically and have all decisions documented. Proof and evidence for calculations is really important. All this is backed by factsheets, examples of use in physical case-studies, videos, and a forum where he can connect with people that have experience with specific type of systems.  **Desirability** – Drag and drop of technologies or build a system by clicking on a template would be very helpful (albeit more advanced than a simple online compendium like in the IHE seminars). Each choice should give short information. Having a better algorithm to improve the current procedure of designing on the basis of spreadsheets with info and empirical formulas based on experience. It should be more efficient to get more accurate designs. Renjali is not good at reporting information, since he is mostly technically trained. He would like to make his reports and deliverables more attractive by using templates. More information on the sector would help him to better play around with novel solutions. At the moment he tries to be as conventional as possible. With more information, he can start to challenge current designs. As an engineer he likes to work with certain level of abstraction. If the tool can do this for him it is extremely valuable and fundamental from an engineering point of view.  **Accessibility** – He wants to be able to show the tool and have it ***accessible to all people in the sector (2/3)*** without making them confused. Simplicity, no registering required. Open website within a few clicks and use it straight away. Process of getting into tool not too complicated, because then he gets distracted. He doesn’t want to fill in a lot of technicalities for the tool to be able to work. | | | |

Planner – “Benjamin Aubert”

The following is based on **6 interviews** with planners in the field of urban sanitation. We define a planner as a (governmental) official in charge of developing (new) urban wastewater management plans. The person is responsible for the compilation of sanitation technology and sanitation system before these are implemented in a local context. This involves stakeholder elicitation, field visits, selection of appropriate technologies, and enabling environment assessment.

For those interviewees that have given similar answers, we have added (n/m), indicating the stringency of an aspect. For example, when two interviewees indicate “local stakeholders” as being important it would look like: *local stakeholders (2/m).*

| General  **Benjamin Aubert**  A planner aiming at bridging the gap between local and global practice on the basis of expertise in multiple countries.  “To implement a sustainable system it is crucial for people to u**nderstand the *‘sine qua non’ (bottleneck) conditions (2/6)*** for the system to work”; “***it is important to go beyond the technical aspect and think more systemic (2/6)”;*** | | | Photo |
| --- | --- | --- | --- |
| A quick take on  Benjamin is a 56 year old civil engineer, born in Chili and working in the water sanitation sector ever since he finished his studies. He has a fundamental drive to help people and enjoys challenging (Wicked, as he defines them) problems. This is exemplified by the variety of positions throughout his career. He first started working for a consulting company best known for the advice and development of water sanitation solutions in developing countries. Clients included UNICEF and the Swiss Development and Cooperation. As a member of the Swiss Humanitarian Aid he worked in WASH, logistics and the environment group and was linked to policy development. He has a strong interest in nature and likes to be in the field to see what the local implication of a new system actually entails. Therefore, when time allows he visits the areas that he works on. This allows him to do his own assessment, get a feel for the project and cross-check information. These projects are distributed over Eastern-EU states, West-Africa, India, Latin America, the Middle-East and Central-Asia. At the moment he works as a Program Director, based in Germany, responsible for managing innovative community-level planning and evaluation of rural and urban projects. | | | |
| Goals and customer jobs  Benjamin makes decisions based on sustainability aspects, technologies that are locally known, space, local capacity, ***affordability (3/6)*,** and ***management capabilities (2/6)*.** In doing so he closely follows the steps as outlined in ***CLUES (2/6)*,** SANITATION 21 (although he finds the newer version is too fluffy), and Community-led Total Sanitation*.* However, a multi-criteria approach to tools and frameworks has his preference, where he borrows bits and pieces that work best depending on the situation. He specifically focusses on the evaluation and development in terms of technology selection for resource recovery. In the basis the answer should always be “will it work?”. To this end he uses the ***Compendium of Sanitation Systems (2/6)*** and excreta flow diagrams (SFD’s) to help structure and communicate. In the last 10 years he tried to get people to consider resource recovery in the planning process by tools and decision-support frameworks. In the main, he connects with sanitation and environmental engineers in his planning capacity. Furthermore, he collaborates with organizations as the SDC, GIZ, SECO, World Bank, BGF, Oxfam, Caritas and Helvetas. | | | |
| Motivations  Realising that it is needed to consider the quality aspects, particularly those that relate to the environment from the beginning of the planning/emerging of an urban context. To help foster a ***paradigm-shift (2/6)*** *towards* ***mixed service delivery (3/6)*** at the higher level *(e.g. UNHCR)*. He wants to ***get everyone to work towards the same goal (2/6)*** in the rapid and unknown growth of urban situations. And he finds it crucial to consider integrated water resource management from the beginning in developing countries. Sanitation is a very relevant field with many needs. Unfortunately he sees that many projects do not lead to the expected outcome, resulting in large mis-investments. He can’t bear to see people living in undignified situations and rather solves the primary needs instead of having to work on further increasing the quality in developed countries. | Attitudes  People start to believe in the power of natural processes, closing the loop, circular economy. There is a shift of decision-making from the expert level to the end-user. This is increasingly more based on tools that incorporate evidence, costs, and context-specific design of systems. To do so, there is more monitoring and data collection needed in both developing and developed countries. ***Technical options and approaches are a small component* *(4/6)*** and should be linked to larger visions so that solutions can more easily be shared and negotiation processes become easier. Specifically, how to connect and upgrade this patchwork of public and private systems (technically, physically and institutionally). It is important to understand the thinking of people working for e.g. the Worldbank group because these organisations provide the necessary financial support. ***Interdisciplinary (4/6)*** and peer-to-peer communication skills are very important. In the field he sees experience, having questionnaires on hand (on a device or paper based) and using the Compendium to check information as important. Because decision making typically focusses on those systems that are known, we mainly end up with small-scale FSM conventional systems in emerging city context. This should be integrated with the compendium that focusses more on natural systems. Because FSM is booming and it’s only 10 years old, both approaches could learn from eachother.  He would like to see a more standardized approach across the field. Better degree of education, freedom of expression and participation. Showing what is possible. Awareness raising. Learning by seeing. Tools alone are not enough: we need to connect to local practices and evaluations. Create learning loops. Resource recovery is not always needed (e.g. because the soil is of high quality) so it is important to avoid misleading people with such solutions | | |
| Pains  Challenging to convince/build trust with people, circulation of negative thoughts. Lack of creativity, ***lock-in of sewered technologies (3/6)*** because these are robust, *seen as* ***golden standard (2/6)*** and expertise to implement and operate these systems is there, ***limited time (3/6)*** and the resulting difficulty to go in depth, further hampered by being an outsider. Too much reading material. Having to know which tool to pick is difficult for practitioners. A system builder considering all options is missing, specifically there is a *gap between planning and* ***implementation* *(4/6)*** including the ***costs (2/6)*** and not having the skills to provide adequate numbers on the implications of preliminary options. There are few good tools around (they miss the “meat on the bone”, i.e. there is no proper toolbox for each step so we end up with a partial output and it is not clear how to connect this to the next step) and to know prior if they are helpful in a particular context. Nobody thinks about sanitation at the field-level (contrary to EU), it is difficult to get actors on board (***poor so why worry about sanitation (2/6)*)** and convince them to build something permanent. High turnover of people in the funding field. Feeling to start from beginning all the time. ***Receiving local feedback that we are trying to peddle systems for us to make profits or test -> Skepticism (2/6).*** Possible interference with other parts of the planning process. Reluctant to rely on platform only, for design of system engineer needed. Decision makers usually not experts. People stay in position of receiver, instead of taking action themselves. Government decides. Mental and regulatory frameworks do not allow to think outside of the box. Some systems are not allowed, even sceptic tanks! Thinking in tables and algorithms is already quite advanced, it cannot be used by general public. Resistance to help because stakeholders don’t see the need/ do not see themselves as developing countries.  *-*  No plans, no data, no maps, corruption. City-planner not knowing about planning. No point in planning because framework (institutions, etc.) not there. | | Gains  ***Case-studies and implementation (5/6)*,** show-case technologies with demonstrations, talk to local leaders first, start with technologies that are as close as possible to what is known. ***Access to information (2/6)*** and understanding of the context. Dialogue, networks and regulations (national or international e.g. by the World Bank). Standardized designs, cost estimations and best practice. Implementation background would help. The benefit of a tool should be clear from the start including what inputs are needed. Straightforward references explaining technology including basic designs and implications. Mobile labs to deliver ***evidence* *(2/6)*** in terms of performance, energy use, costs to get donors on board and higher-level support. Having local technical expertise on site that tells me what is possible. Advocacy by case-studies. Regulations of technical specifications per country that need to be followed in the design.  In his role as FSM planner he uses knowledge-gap matrices and focus-group discussions to gather the unknown details which allows him to appropriately develop the business model. | Expectations  “Must-haves”  The translation from technical results to ***understanding for the general public (2/5)*,** based on the ***enabling environment (3/5)*** (CLUES) It should ***convince students and municipalities to use the platform (2/5).*** Having resources (a la ***Compendium (3/6)*** and SuSanA) and case-studies online. But it should outperform the Compendium. Different levels of knowledge entry and bridge the gap with implementation.  Main platform functionality  ***Case-study information (5/6)*** and ***relevant contacts (3/6****)* to talk to. A decision making tool for the assessment of well-defined sanitation technology and systems options and to narrow down the scope of options. |
| User experience  **Usefulness** –. It should be accessible both in the office and on the field. Information should be available on the basis of ***peer-to-peer/grey-literature exchange (5/6)*** on ***case-studies (5/6)*** especially on novel technologies*.* It should show what the added value is, specifically what the costs are compared to the Business as Usual BAU (including externalization of costs). Good promotion material, showing physical improvements.  **Usability** – it should be user-friendly, intuitive, and simple, but not a black-box. Display the information on what is needed to create the enabling environment alongside the technical solutions in digestible quantities (in view of time). The results should be detailed enough to do a sustainability assessment, the consultant can start designing the system, and potentially link it to case and country-specific options. Linking questionnaires on smartphone. Electronic guidance to dig in at level that is appropriate (full planning vs checking if something is feasible). Tool should take user from appropriate point and assess how much time is there to spend and what level of detail should be. Generalize the tool so that it can be used by people who think differently.  **Findability** – Subscription to mailing list to know where to look when question comes up, even though he does not always read it, it helps to remind what is available.  **Credibility** – Decisions are made on the basis of ***evidence (3/6)*** and the compilation of information that comes from community itself. The tool should be linked to how it is used in ***physical case-studies (3/6)*** that show what is achieved by using it (FSM toolbox is a good example), and what can be done with that information. A partial output makes people question the overall validity. It should enable consultants to go further with ***confidence (2/6)*.** Show publications, have information from outside translated into local language. Send data on proposed systems to Eawag for validation  **Desirability** – have concrete arguments and numbers for technology options. Toolbox that would interpret legislation, funding, upkeep, construction of the proposed solutions. And it should answer if it works by 1) where are we now, 2) where do we want to go, 3) ***how to get there (2/6)* *Short video-clips* *(2/6)*** a la FSM (instructions for tool, explanations of case-studies: what was the problem and how is it solved?). 1. Assessment, 2. Selection of options. 3. Check-list to know what is needed for the enabling environment. Computerized decision support system – recommendation of tools based on the situation the user is in. Tool should provide information as to how address enabling environment gaps. Top-notch graphical outputs that are important for effective communication, presenting content that can readily be adopted in reports. Tool as catalyzer to bring many settings to higher degree of awareness. Not only experts, also DMs, locals, planners.  **Accessibility** – ***The tool should be accessible to everyone, not just the experts (2/6),*** including the data. Different levels of entry to use the tool. Compendium useful for those with training and time. For others provide opportunity to join in on online workshops. The platform should remain open source. | | | |

Researcher – “Winston Patou-Agye”

The following is based on **6 interviews** with researchers in the field of urban sanitation. We define a researcher as a person performing research in the field of sustainable sanitation technologies, policy development and social sciences working on design, compatibility or improvement of novel and current sanitation solutions. This includes academia and technology developers.

For those interviewees that have given similar answers, we have added (n/m), indicating the stringency of an aspect. For example, when two interviewees indicate “local stakeholders” as being important it would look like: *local stakeholders (2/m).*

| General  Winston Patou-Agye  “Finding alternatives to show how we can deal with wastewater in the future”  “Doing cool stuff” | | | Photo |
| --- | --- | --- | --- |
| A quick take on…  Winston is a 34 year old assistant researcher in urban sanitation. He has a background in environmental engineering and sciences. He worked for 10 years on urban sanitation, mostly Strategic Environmental Planning where he conducted fieldwork mainly in Malawi. He helped develop guidelines (such as CLUES and Sanitation 21), publications, trainings, small implementation projects, and did additional research in India. Although he is a researcher, he is slowly transitioning to the implementation (planning/engineering) side, aiming to apply what he has learned over the past years. | | | |
| Goals and customer jobs  In his research he focusses on community involvement in sanitation planning and resource recovery in decentralized sanitation solutions. His research focusses on India and South Africa, but the outcomes have general relevance. Most of his time is spend on research, but understanding of and collaboration with policy makers, stakeholders from industry, research, practice and specialist groups and networks such as SuSanA, and the International Water Association is equally important. It helps him to develop innovative suggestions for novel technologies and to facilitate space for these to emerge from the research context.  Writing research publications is undoubtedly important to create and facilitate knowledge. It is needed to convince, and create momentum with the international community which is the ***ultimate environment for publications (2/6)*** to be placed and taken up. They are crucial for the next step, since it builds and strengthens the case, especially for those working on the interface of policy and science. He conveys the knowledge during conferences and meetings, but mainly in personal interactions. Further he supplements pre-developed list of objectives with community-based objectives to develop refined screening criteria as input for planning software.  At the start of a new research project he generally first decides on the ***scale and level of centralization of the system (3/6)***, the area and objective of intervention. Then he goes out there to talk, and ***understand the key challenges (2/6)*** that need to be tackled and then goes through the options by structured methodology and tools. During field-work he collects data. Then, he checks what has been done already through the internet and discusses possibilities, implications and assesses what can be connected during personal meetings with experts. In addition, about 20% of his time is spend on talking to people outside of academia. Being there, answering to invitations and giving presentations is very valuable in long run. This helps him for example to understand better what can be done within the boundaries of funding schemes and the power dynamics of the stakeholders. Difficult trade-offs are always costs and land requirements, because decisions are usually made on the basis of investment costs, not life-cycle costs. Data, technology, religion, ways of thinking, lack of centralized planning, climate change and corruption are important considerations. After this a tool can be used to identify technological solutions in light of also the non-technical considerations such as the legal framework and topography. | | | |
| Motivations  He sees his research as an attempt to slow the apocalypse. He finds it ***shocking to have this miserable urban sanitation situation still in 2020 (2/6).*** He wants to make ***cities and residential areas more livable (2/6)***, and finds it better to work as a researcher on the root cause of a problem.  There are many indicators where we should start to think different. Very pressing are the alarming **deteriorating *environmental factors (2/6).*** The latter is most clear in the fact that people still use drinking water resources to transport excreta. He feels that something must and can be done. Here he finds the integration of ***different disciplines (2/6)*** most interesting and complex, and enjoys having people around him with the capacity to think ahead and look forward. He is motivated by what can be caged in urban water management to stay compatible with the future. This requires the continuous input of new ideas and to move forward. The installment of more lighthouse projects, and getting technologies higher up in technology readiness level could help to do so, specifically because practice lacks about 10-15 years.  He hopes to support a paradigm shift to a modular approach which entails the larger acceptance and visibility of option space among the wider society. | Attitudes  He does not use tools much but uses whatever solves the problem.  He does not believe that tools support engagement, but that the existing situation needs to get worse before it gets more on the mind of people. Engagement can be further fostered by the ***inclusion of other disciplines (2/6)*** (e.g. stormwater management, irrigation, trash-collection, and infrastructure). However, he also believes that the more people grow up with internet, the quicker they would resort to the use of web-based tools first. This being said, he was impressed with the first version of the mock-up, because it was intuitive.  Main considerations here are capital vs investment, long-term planning, and operation and maintenance.  He thinks a two-day workshop is needed for the tool and one week to explain different systems, although he believes that going from a focus on just pit latrines to over 40 options is hard. The mere idea of having a choice at all is in many contexts very new.  He believes that the situation does not receive the attention it deserves, and is hampered by the lack of transparency and accountability in the process. But hopes that people will start to understand better that there are multiple options to be evaluated based on clear and transparent criteria.  -  We cannot change cities, but we can provide theoretical base by methodology, tools, showing what functions and show what are the gaps.  Best cooperation with people where money is not on the forefront.  Science is a people’s business.  Knowing how to function, and having trust in other disciplines that their outcome is valuable.  Natural sciences vs social sciences approach needs to be bridged.  Iterative learning from practice.  Having fixed position allows for long-term planning and absolutely essential to have fulfilling scientific life.  Knowing who the user base is, is absolutely crucial.  -  Need for more participatory research and contextualization of knowledge.  Continuous cooperation between practitioners and academia to maintain accessibility and understanding. From beginning: setting goals beneficial for both ends.  Money and time would help to do better.  Wouldn’t always trust a tool to function, they serve more as guidelines. Like to look at various tools. Therefor platform could be valuable.  Collaboration with local experts to ensure proper research can be conducted.  ***Doesn’t think much will change quick (3/6).***  Hopes that uptake of technologies will be larger by cross-sector opportunities for collaboration. | | |
| Pains  ***Complexity of topic hard to grasp and address (2/6),*** specifically the ***understanding (3/6) for non-experts (2/6)*** because there is ***not one storyline or solution (2/6)*** that everyone taps into.  Case-specific importance of criteria and local requirements.  -  Hard to involve people in workshops.  Pre-defined objectives not on mind of locals, which makes it hard to work towards one part of a goal without understanding the long-term effects. It is particularly difficult for people to think about what will happen in the future, let alone that politicians have a clue of the practical implications, nor the particular importance of transparency and accountability in decision making. This could be due to ***low skills (2/6)*,** e.g. people do not think in matrices and options and people in government being able to use excel is already very new.  Weak institutions, no overarching plan of procedures and checklists and unforeseen issues leads to slow progress and ***sanitation being low on the priority list (2/6).*** Furthermore, there is no need to think about sanitation if there is enough space outside city, pit-latrines themselves are already seen as proper sanitation and more is depicted as something from “white people”.  Most information paper-based, no-one knows where it is.  Impacts coming more from religion than science.  -  ***Time constraints (2/6)*** (pressure on result delivery) and the balance of delivering quality with a usual ***lack of financial resources (2/6)***.  Usually not interdisciplinary.  Expensive to implement infrastructure  Centralize or decentralize: no good established procedure and checklist yet!  Cost data of unconventional solutions.  Too many engineers with bias and not right skills to address mostly non-technical problems.  -  Describing interdisciplinary work.  Understanding and being able to learn and trust output from other sciences.  Coming up with at least one alternative that has similar scalability of sewer system anywhere in the world.  Open-up engineering possibilities in practice.  -  Disconnect academia and practice – practice doesn’t have access to tools developed. Academia assumes that people from practice have access to what is produced.  Conspiracy theories about new technologies.  Balance quality of work with available resources.  Most research driven by conditions that I do not have control over, such as grants. | | Gains  Availability and high technology readiness level.  Taking into account both up and downstream of chain.  Acceptance and more help from the sector and society  (planners, architects and engineers) knowing about technologies. ***Coupling to trending or salient topics (2/6)*** helps to bring discussions on level with authorities and to feed into legal frameworks and political agenda. This ultimately results in all aspects from the ***enabling environment to be in place (2/6)*** allowing for new technologies and resource recovery. Further it helps to reduce the option space (3 is a lot already).  Having ***time (3/6)*** to spend on doing unproductive things**,** ***resources (3/6)***, flexibility (PhD unflexible, only 4 years)  Crossing disciplinary boundaries.  ***Guided tours and field trips (2/6)*** to show importance of research.  It helps to have an education, being able to communicate and having and engaging with a good ***network (2/6)*** of ***motivated (3/6)*** and reliable experts. They have the knowhow on technicalities and the local situation. Trust is key.  Further it is important to have ***access*** ***to*** ***publications (4/6)***, knowledge, and guidelines/tools with guiding principles/step by step approaches and tools to not forget important things (best practice, do’s and don’ts) and be aware of the complexity.  In terms of tools and approaches he uses ***CLUES (2/6)*** (tool D17.1), SOAD (decision making tool), Sanitation 21 and the Compendium of Sanitation systems and Technologies system templates. In addition he uses software such as R, Julia, Python, SWIM, Epanet, GIS, and Urban Beats (a development platform). | Expectations  “Must-haves”  Outreach for practitioners.  ***Pilot projects (2/6).***  Collaboration with industry.  **-**  Should be better than what is happening now.  -  Good guide, economic criteria.  Generalized checklist for local requirements.  Current and forecasted situation: technical and socio-economic, areas with priority.  -  Qualitative assessment of how new technology can be integrated in sanitation system.  Practical reasons. Transition planning, what kind of options are there to move to the next step.  Linking engineering properties with planning (so far non existent.)  Bridging traditional process/system engineering and transition in developing countries.  New, surprising and useful results.  Magic of expert use needs to be clear for those that are ready to make money with it and are not shy to dive into deeper level a la Mouse.  Being generic enough to also be used in five years time.  -  Provides information that goes beyond what can be published (stakeholder opinions of processes and the like)  Should tell needs of specific stakeholders.  Should show me research papers, policy briefs, when I search for it.  Main platform functionality  Collaboration  Output of products and service implications (capacity, *capital costs (2/6)*, *O&M (2/6)*, management and monitoring, who to contact in case of problems).  -  Learning, planning, engineering.  -  Learning.  User-board where users can ask questions and help eachother.  Courses that can be joined. |
| User experience  **Usefulness** – Guidance on how to deal with data gaps, e.g. expert, assumptions, empirical values, rules of thumb. What is crucial data, what does it mean to get this data.  If it’s good and giving relevant hints. Do’s and don’ts, checklists.  No weak data, so far the cost aspect is missing in most tools.  -  Should be better than what he can come up with intuitively.  Should get a lot of things done, e.g. SWIM generic tool to model hydrodynamic situation in catchment area.  Should be able to reference to it, to be able to transfer, can be properly references to use in a publication (it should thus describe to other people all details without me needing to do it). It should be transparent enough to know how it works and where to get it.  -  Should make work easier, instead of having to use multiple sites or diving into databases.  **Usability** – Output of different system configurations as basis for discussion and shift research focus/widen view.  -  Not 300 parameters required. Gamification.  -  Should keep fulfilling (new) needs that are out there.  **Findability** – Mailing list  **Credibility** – ***Pilot (lighthouse) projects/ case studies where tool is applied and proven to be useful (2/6)***  -  want to be confident he’s doing the right thing.  Understand clearly what it does (no black box), calculations accessible, need to be able to run these himself. Transparency (clicking further to details) to build trust.  -  It either works or it doesn’t. Does it get the job done?  Everybody knows it, high confidence in correctness of results. Open source. Can be developed further.  Transparency, reliance, software can be accesses by others  Full scientific documented backing.  -  **Desirability** – Guidelines to creating environment/demand. Introduction to planning process for people new to the field.  -  Support for different storylines. Showing what opportunities are (needs from practice, industry). Include research questions.  Providing decision support not limited to technology, but based on politics and legal frameworks. A scientifically endorsed MCDA approach for identifying and assessing alternatives. Structured approach would therefore be helpful to indicate why decisions are made.  -  Small modules (without being bombarded by the whole!) Quizzes: can this connect to this? Showing benefits and downsides.  -  Manuals to be used in different contexts (translation issues result in different meanings in local context)  World-map with case-studies indicating approaches and tools used. Clicking should give summary of project and outcome, indicating successes and challenges.  Glossary with translation of terms. Visuals.  Contextualize gap between Global North and Global South.  -  This is helped by more efficient online ***collaboration (2/6)*** with less emails.  **Accessibility** – Structured information trees, simple layouts, easy navigation.  -  Computer, office. Split user experience to engage with different backgrounds (problem: getting storylines back together)  -  Internet is patchy and expensive. Offline, on ***PC (3/6)*** and ***mobile phone (2/6)*** (mainly Android). App-based data collection that can be uploaded directly into his account on the platform. Step-wise and incremental, build understanding.  Should not feel super technical. Decision tree with yes and no answers. Visuals. Accessible for people speaking Arabic.  -  Different levels of complexity. Want to be able to produce a result within 10 minutes for a first draft idea. Then when creating more detailed idea ability to transfer and run it from my own server.  Gamification (levels beginner, advanced, expert). From more guiding to less guiding. Capture everybody using it not just expert.  Easy entry into software with videos, point and click. SWIM very complex but can be used within half an hour. | | | |

Teacher and trainer – “Margot-Luise McDevin”

The following is based on **6 interviews** with teacher and trainers in the field of urban sanitation. We define a trainer or teacher as a person in charge of training people that are involved in relaying the basic functioning, pros and cons of sanitation systems and technologies for O&M purposes and/or urban wastewater management development.

For those interviewees that have given similar answers, we have added (n/m), indicating the stringency of an aspect. For example, when two interviewees indicate “local stakeholders” as being important it would look like: *local stakeholders (2/m).*

| General  **Margot-Luise McDevin**  “Real goal is to get the audience to do something better because of a training.”  “Ecological sanitation is another area, it is like changing someone’s religion” | | | Photo |
| --- | --- | --- | --- |
| A quick take on  Margot-Luise is 39 and originally from Ontario, Canada. Her studies in civil engineering were focused on septic tank management, regulations and control. First she focused on the US, and then shifted to development when working three years for Eawag/Sandec as a research assistant and later as project manager. As a young consultant, she has been involved with the sanitation campaign in Bangladesh. She helped reduce the open defecation from 42% to almost 0% from 2003 to 2015 by organizing conferences and the sanitation forum, now seen as learning events for other countries. These joint-force conventions have convinced governments and resulted in constructive collaboration with policy makers by setting the agenda.  She owns a patent on the design of a waterless low-cost urinal system, that she developed together with the International Resource Centre, Delft. It was deemed the best innovation for low-income communities in development countries. Recently, she has been involved in the development of a decision tool for CAWST (a US-based education and training center) on how to get to smaller set of discussion options for toilets and containment. She worked on many sanitation projects in low-income urban settings when she worked for Unicef. Here she was mainly responsible for community engagement and the development of a teaching module on sanitation. | | | |
| Goals and customer jobs  At the moment she is lecturer in a rural development institute, ***teaching governmental officials (2/6)*** on several technologies (sanitation and solid waste), and the regular cycle of planning, implementing, and evaluating. Although the main focus is on rural, her focus increases on sanitation in the peri-urban and urban context, and rural she does more trouble shooting. Part of her job is ***capacity development (2/6)*,** development of course content and indicators to be used for SDG, as well as the training of trainers in the field of sanitation. She finds it important to teach on the broader concept: City Wide Inclusive Sanitation (CWIS) approach, foster new ways of thinking and data collection, and not only focus on the technical side of the solution. This is further exemplified in her efforts in developing national sanitation strategies, the WHO city sanitation planning and being involved in Faecal sludge management.  At the local level she interacts with municipality mayors and at project level she does ***pilot (2/6)*** with local supervisors, private ***consultants (2/6)***, ***engineers (2/6)*** from engineering departments, ***policy makers (2/6)***, scientists, PhD students and ***master students (2/6)*** from universities such as the TU Delft and TU Berlin as a guest lecturer. In the projects she is responsible for the evaluation of new technologies, piloting and project implementation.  For a new project she first tries to understand the needs, requests, and competency gaps of the audience as well as their learning preferences and motivation gaps. Then she conducts literature reviews and consults partners to see what trainings are available, after which she finds government support/donors. At last she does a trial run to get feedback and finalize the material for delivery. In order to make sure that her content delivers, she finds it crucial to follow-up with the participants and support them by suggesting particular tools if they are struggling after the training. To support people effectively she therefore introduces the tools during the teaching or training to make people already accustomed to what is there.  Some of the aspects that she commonly treats are feasability, costs, required workforce, public or community systems, life-time of tech, user-friendliness, integration into existing technology, outcome measurements, and stakeholder engagement. | | | |
| Motivations  ***Contribute to development (2/6)*** and the promotion of technology to teach about a different context and hopefully affect people’s lives by aiding the reduction of open defecation.  The more people work on this, the more it comes into the picture. Therefore she is motivated to train the next generation of engineers, public health officials and other relevant stakeholders about urban sanitation. Specifically to train them to think about options.  In addition, she enjoys to address problems and be continuously on top of something new that is ***interesting (2/6)*** and inspiring to her.  Very few people think about sanitation. | Attitudes  She sees sanitation systems as being deep in technical knowledge, therefore a strong ***technical background is needed (2/6)*** to understand and work with them. Because of this in-depth knowledge, it is important to address the ***gap between design (research) and implementation in the field (3/6)*** which involves ***politics (2/6)***. The ease of access of a platform for political leaders and policy makers could help avoid researchers/academics to have to relay this specific information and fast-track momentum in the field. Furthermore, it is important to learn lessons from other countries and see sanitation as one of the services next to other urban services.  Planners usually sticks to options that they know. These options are merely based on ***costs (2/6)*,** maintenance and a only a few main parameters that are considered the important design criteria.  It is needed to demystify technologies, engineers now follow handbooks for dimensions, etcetera, but for sanitation this is not available. Engineers should understand decentralized treatment plants, but this change is slow.  During her trainings she learns by doing in didactical and pedagogical skills and appreciates all inputs that she can get, especially tools. To help her she uses tools (such as the FSM toolbox and videos), of which she finds the exposure during trainings fundamental. She notices that if tools are only directed to INGOs and Development organizations, they might not be very helpful however, because the real audience are those that actually do the planning. Moreover, the ***use of a tool is often quite different from what is envisioned (2/6).*** She generally sees that tools are being used less and less, unless their use is required by e.g. the World Bank.  She deems online/blended ***online/blended (training, teaching, worskhops, conferences, seminars) (2/6)*** beneficial as it reaches many more people simultaneous, however she is not yet convinced about replacing the personal atmosphere and real-live discussions. Moreover, she is afraid that snappy online inputs leads to consumers losing their ability to oversee the larger picture.  In general she finds it important to refrain from assumptions, but asks questions and focus on finding possibilities for partnering to find answers in a local situation. Better coordination, increasing government priority and allocation of funds helps her to get to the right users (especially those that are unexperienced), make them understand the real purpose, and how to get started and how to judge the results.  -  Shocking students into reality with worst case and best-case scenarios.  -  If design not done by engineer who will manage system in the field. If he doesn’t understand the system it will fail.  -  Virtual reality meetings. IT-based systems (internet of things) to monitor, more remote managing.  Consider how to involve civil society! | | |
| Pains  Even at highest level, the ***appropriate technical and design knowledge (2/6)*** is missing. Even those that approve systems do not know the systems.  people are tired and sewage is easier.  Missing of appropriate interfaces for ecological sanitation.  Engineers in the field find it difficult to plan.  Engineers and planner not go through full menu and options like envisioned by CLUES. The older the more narrow-minded. Harder to accept new perspectives.  Lack of knowledge of options.  Building standard systems without regard to case-specific dimensions.  ***Time and resources (2/6)*** ***to find and get into the right tool (3/6)*,** even Compendium.  Texts are difficult, tools very contextualized. When manual looks hard people forego reading it.  ***Engineers are very self-confident (2/5)***, using tool means accepting someone else’s view, and do not like to sit down and discuss with other people.  Compendium only takes me half way.  -  Modify module every year because of new technologies.  Asking others to access literature for you.  Lack of labs, databases slows down.  Not having money for field excursions -> teaching too theoretical.  -  Older private consultants that work in field that I do not know well. Challenging to understand mind-set of audience. Master students I do understand because of my background.  -  Training content requires 10-15 days for successful delivery.  Representation of relevant persons in training.  Mayors not convinced enough because other priorities.  Matching requirements to budgets, availability of land. Urban rapid and unplanned growth. Mismatch with other services already available.  -  How to stay connected and support participants after training.  Remote delivery of service: band-width of devices, and technologies used to access trainings.  Most tools not well visible/usable on phones.  Tools mainly designed for NGO’s and development organizations.  Many tools complicated output.  -  Costs, transportation, permission, time. Rich vs poor understanding of connection sanitation and water.  Thinking in trade-offs is very new in developing context. At present: right, wrong and memorizing. | | Gains  ***Being associated with various projects and consult stakeholders/expats (5/6)*** and ***partnerships with*** ***Eawag (2/6)***, ***Concad (2/6)***, CSE India***.*** The Hygiene Hub, conferences and meetings and being involved in pilot studies.  Being able to use frameworks and tools supported by UNHCR, UNICEF, and the World Bank e.g. Community-Led Total Sanitation, and others such as the ***Compendium (2/6)***, SuSanA, ***CLUES (2/6)***, City-Wide Inclusive Sanitation service chain, ***FSM toolbox (3/6)*** videos**,** ***Sanitation 21 (3/6)***, task based training to have actors experience stages in ***City Sanitation Plans (2/6)***, Shitflow diagrams, and Inclusive Sanitation in Practice. Then it is important to understand the didactics around how adults learn. And have internal and external evaluations (ISAN UK) of the trainings. In terms of software NOMA, Excel, Stata, SPSS, AtRisk (quantitative) en EnVivo (qualitative).  ***Trainings should relate to national development plans (2/5)*** to ensure ***endorsement from government-level (3/6).*** This results in a mind-set change, regulations, and planning for urban development that ideally move in a more or less similar timescale. Here the relevancy and benefits for target group by linking the modules to day-to-day activities, creating understanding of efficiency, importance, and political popularity is crucial.  Literature review and access to publications, practical experience and degree of know-how, ***surveys (2/6)*** or ***interdisciplinary approaches (2/6)*** such as GALS approach (focus group discussions), field-trips, ***lab-work (2/6)***. Resources (facilities to go online in times of crisis (COVID))  Good and motivated team and working environment, clear communication and clearly assigned roles, responsibilities and guidance.  Understanding sanitary behaviour and more advocacy for ecological sanitation.  Emphasis on teaching other professors, lecturers and engineers. | Expectations  “Must-haves”  Platform should host ***practical experience/case-studies (3/6)*** to work on. It should have a clear advantage over multitude of other platforms by ***providing umbrella (one-stop-shop) (3/6)*** of a knowledge hub. If information cannot be given by the platform ***links should be available (2/6)*.** For example, an overview of all tools, needed to collect data for bidding and winning projects should be provided.  It should be directly apparent that results and access to new information is ***effective and less time consuming (2/6)***. For example, data collection should not be too difficult and less resource intensive than what is available now. This could be helped by small videos explaining the tool and applicability to ***different stages/scales/spatial levels (2/6)*** of the process.  The results should translate into the design and implementation phase, i.e. answering the what is next.  ***Comprehensive (2/6)*** prepackaged ***training modules (3/6)*** should be readily available.  s  Main platform functionality  ***All stages of the process cycle (2/6)*** should be integrated.  Specifically learning, teaching, ***planning (2/6)*** (narrowing down the option space), and design should be treated and made simple. This can be done by providing an ***overview of various*** ***thematic areas (3/5)*** and approaches to understand thinking/philosophy behind these. |
| User experience  **Usefulness** – Compendium into computer based package to make identification process easier and know what parameter to look at. ***Provide design options/drawings (2/6)*** that are directly applicable in the field alongside the selected options. It should host a guiding to the entire process. It would help if the platform or tool could speed up meetings, discussions with people, and creating consensus. Here knowing what the broad options are and which are applicable to local situation would be huge advantage. Further the possibility to follow-up and access the platform with links provided through Whatsapp would be super helpful, it would also be useful if it is available as an app.  **Usability** – Pictures and ***videos (2/6)*** on engagement and how to judge outputs should be usable by municipalities. Further it would help if the platform should be interactive for example on the basis of GIS denoting places/thematic areas on maps that open more details upon clicking.  **Findability** – Search button is important and/or a small survey eliciting what you are actually looking for should suggest ***relevant content (3/6)***.  **Credibility** – When engineers come to the platform, the planning should be automatically taken care of. Provide benchmark, info of where the platform has been used, ***link to case-studies (2/6)*** so people can see how well it works in practice, raising credibility. She likes the design of the earliest version of the Mock-up: here are the options, these are the tradeoffs. Inputs vs performance vs operation cost, etc. These are some options that you should consider further, with some features highlighted that might have to be looked into (this should be very clear from start).  **Desirability** – People should see value - the materials to teach, learn and plan should be made attractive. At best it should host two-hour effective designed training programs, improving skills of trainers. A shorter time is more valued over longer time-involvement in online trainings. The modules should contain clear planning processes, review of existing systems, and resource planning. It should indicate actual interventions and needed timeframes, as well as action plans supported by immediate visual output.  The interface should not be too crowded, but only present key/broad thematic areas. ***It should be nested (2/6)*,** i.e. like a tree diagram: the more detailed the more you go down. It would be great if it links to the Sustainable Development Goals and works in a Wikipedia format by linking different, related, terms.  **Accessibility** – It should be free and available ***offline and online (2/6).*** A small introduction and guideline should allow for easy access, understanding (of how to fit the platform in training and planning process), navigation, and obtaining training materials from the platform ***for all (2/6)*** (benefitting the primary target community/municipality as well as experts/consultants). Training modules should be accessible ***from any device: desktop, laptop, tablet (2/6) that is operated with low bandwidth data from cellphones (2/6)***. | | | |
