# Supplementary material for: Closing Water and Nutrient Cycles in Urban Wastewater Management: How to Make an Academic Software Available to General Practice
Source: Circ Econ Sustain. 2021 Jul 15;1(3):1087–105. doi: 10.1007/s43615-021-00073-6 (PMC8679645; doi:10.1007/s43615-021-00073-6)
Supplement: Supplementary file 3 — (DOCX 31.1 kb) [file 43615_2021_73_MOESM3_ESM.docx]

Closing water and nutrient cycles in urban wastewater management: how to make an academic software available to general practice

Johann S. Schuur^a,b^ and Dorothee Spuhler^a^

(0000-0002-0924-2319, 0000-0002-1379-6146)

Corresponding author: Johann S. Schuur, [jschuur@ethz.ch](mailto:jschuur@ethz.ch)

*^a)^ Eawag, Swiss Federal Institute of Aquatic Science and Technology, 8600 Dübendorf, Switzerland.*

*^b)^ ETH, Swiss Federal Institute of Technology, Institute of Science, Technology and Policy, 8092 Zürich, Switzerland.*

Description of content: This file contains a filled out framework (Table 1) that brings together the analysis from the personas with the lessons learned from practical application of SANTIAGO. This leads to specific design choices for the development of SaniChoice.

**Table 2** Full representation of the framework as depicted in the paper (Table 1), indicating specific understanding of each user category on the basis of personas including main needs and expectations, linkage with requirements arising from practical applications of SANTIAGO, and resulting concrete desires from practice that can be translated into design specifications of SaniChoice.

| **Table 2 cont’d** | | | | | | |  | | |  | |
| --- | --- | --- | --- | --- | --- | --- | --- | --- | --- | --- | --- |
| **User understanding extracted from personas** | | | | | | | **Requirements extracted from SANTIAGO field-testing** | | | **SaniChoice**  **design and architecture** | |
|  | | **Capacity builders** | **Engineering experts** | **Planners** | **Researchers** | **Trainers and teachers** | **Use cases**  (1 - 3) | **Expected added value**  (1 - 5) | **Learning targets**  (1 - 6) | **Synthesis** | **SaniChoice provides** |
| **Practitioners (P)** | **Tasks** | Facilitating the customization of existing knowledge (gaps) to context specific project proposals and mission statements. | Design and presentation (to government officials) of simple, smart, and user friendly treatment plants and assessment of technology performance.  Analyse the current situation of technologies, practices, and costs. | Making planning decisions based on sustainability, spatial, affordability, and management capability aspects.  Cooperate with engineers, and international organizations. | Writing publications.  Field-work.  Collaboration with policy makers, (local) stakeholders from industry, specialist groups, and networks. | Lecturing governmental officials on sanitation technologies and the process cycle of planning, implementing, evaluating.  Development of capacity, course content, trainings and indicators for the Sustainable Development Goals.  Interaction with supervisors, private consultants, engineers, policy makers, and researchers. | NA | NA | NA | NA | NA |
|  | **Thinking and attitudes** | Believe that solutions should be embedded in the political, social, and economic context thereby going beyond technical considerations alone. | Think that adoption of tools can form instructive role if simple and understandable by people using systems and technologies. | Try to intgrate resource recovery (if appropriate) by standardized tools and frameworks that are more focused on the end-user and include system evidence, costs and design.  Understand that the technical solution is a small component and that interdisciplinarity is crucial. | Believe that tools do not support engagement but inclusion of different disciplines does.  Don’t think that there will be big changes anytime soon, but hope that collaboration with local experts and uptake of new technologies will ensure that proper research can be conducted. | Finds that sanitation systems require deep technical understanding, therefore it is important to address the gap between research and implementation and involve politics.  Planners usually stick to options that they know.  Application of tools is often quite different from what is envisioned. | NA | NA | NA | NA | NA |
|  | **Motivations** | Enjoy bringing people from different backgrounds together, and create understanding in a highly relevant field. | Development of sustainable technologies to contribute to a circular economy and simultaneous working on complex (technical) puzzles involving many different sectors. | To help foster a paradigm change to the support of alternative sanitation solutions by large organizations in order to reduce mis-investments and to get all actors to work from a similar basic understanding. | The deterioration of environment, understanding of the shocking sanitation situation and a desire to make urban areas more liveable.  Support of paradigm change to modular approaches which entails increasing the visibility of (novel) technologies.  Collaboration with different disciplines. | To help contribute to development and promotion of sanitation technologies in order for more people to work on the subject and hopefully affect people’s lives for the better and to be continuously on top of something new. | NA | NA | NA | NA | NA |
| **P needs** | **Context understanding** | Want to be able to relate information to regional, national, and international based directives, projects, resources, and organizations.  Underline the need for case studies (involving the tool) on technologies and systems. | Want to learn from documented case studies on technology and system options.  Want to have the possibility for peer-to-peer knowledge sharing  On the entire sanitation chain, understandable by the general public. | Want to learn from documented case studies  Require information on performance evidence of possible sanitation technologies and systems, as well as on local behaviour. | Require transparent documentation of previous application of the tool and related advantages and limitations.  Need to understand the scope of the tool.  Scale and level of centralization to which solutions are applied  Key challenges in the implementation of solutions.  Understanding from what organizations the largest impact comes. | Require an understanding of the political dimension, i.e. what is needed for effective implementation.  Needs, requests, and competency gaps of audience as well as learning preferences and motivation gaps. | 1 - 3 | 1 - 5 | 2 - 5 | Understanding is largely governed by case studies and consideration of political/organizational dimension. | Understanding of applicability and success of the tool in a given case study |
|  | **(bridging) planning, design decision, and implementation** | Require pilot projects that involve NGOs, policy makers, and interdisciplinary (local) stakeholders. | Interpretation of results  Provision of complete performance evidence of technologies | Use of knowledge-gap matrices and focus-group discussions to gather unknown details.  Translation from technical results into understanding for general public based on the enabling environment.  Answering the  “*Where are we now”, “Where do we want to go”, “How to get there”.* | By understanding the scale and level of centralization of the system, key challenges in implementation, and boundaries of e.g. political and funding schemes.  Foster engagement by including different disciplines and sciences.  Continuous cooperation between research and practice from beginning until the end.  Linking engineering properties with planning. | Case studies (involving the tool).  Access for political leaders and policy makers.  Linkage to national development plans, various projects and institutes.  Consultation of stakeholders and experts.  Design options and drawings. | 1 - 3 | NA | NA | Users identify interdisciplinary case studies and cooperation crucial to bridge the gap between planning and implementation. Specifically, linkage to (inter) national development plans, guidelines on understanding data-gaps and the level of centralization is key. | Can help create a common understanding for stakehoders with different backgrounds and thereby helping to overcome possible gaps in understanding between the different parts of the sanitation chain. |
|  | **Access to information** | On case-studies, available technology options, tools, and frameworks. | On peers to share knowledge (about case studies).  On the sector and novel technologies to start challenge current designs. | Based on peer-to-peer/grey-literature exchange on case-studies (especially on novel technologies).  Overview of available tools and frameworks. | International publications, policy briefs, and guidelines/tools.  A network of experts (from other disciplines).  Beyond publication context, e.g. stakeholder opinions. | Overview of available tools and frameworks.  Surveys. | 1 - 3 | 2 - 5 | 5 - 6 | There is a strong desire for a *“one-stop-shop”* platform that presents an overview of practices, case studies, tools and approaches used in the sanitation chain. | Mainly planning and decision support in the sanitation chain. |
|  | **Additional services** | Require pre-packaged approaches to planning, workshops, trainings, conferences and seminars, courseras, and Massive Online Open Courses. | Templates for deliverables and reports. | Linkage to country specific requirements and technology options. | Exchange with a user-board.  Small-modular courses (incremental learning). | Pre-packaged training and teaching modules/workshops.  Hosting conferences, seminars.  Overview of thematic areas.  Understanding didactics around adult learning. | 1 - 3 | NA | NA | Users require pre-packaged approaches, templates, and a source where they can connect to stakeholders to help awareness raising. | Although Sanichoice does aid in the three use cases, these user requirement cannot specifically be met by the tool but requires a larger platform. |
|  | **Quantitative (1), qualitative (2) estimations** | (1) of life-cycle costs. | (1) detailed enough such that a sustainability assessment could directly be performed.  (1) sludge quantity estimations.  (2) technology applicability filter. | (2) decision-making tool for the assessment of well-defined technologies as well as narrowing down the scope of options.  (2) checklist to know what is needed to create the enabling environment required for selected options. | (2) assessment of how technology can be integrated into sanitation system.  (2) implication of proposed options with regard to costs, operation and maintenance. |  | 1 - 3 | 1 - 5 | 1, 4,6 | Users want to obtain quantitative and qualitative performance data to narrow the scope of technology options and how they fit into an implementation context considering the enabling environment. | Narrowing of the scope of options based on quantitative and qualitative assessment of (novel) technologies. However, assessment of the needs of the enabling environment are not provided. |
| **P experiences** | **Usability** | Through easy and shareable tutorial videos and written guidance on use of the tool. | By explanatory videos (of applicability of the tool) on the sanitation chain.  Short and concise frameworks that help develop designs.  Answering the “Why”, “What”, and “How” of technology. | User-friendly but not a black box.  General enough to be used by people with different backgrounds.  Differentiated use, e.g. quick checking of feasability and detailed planning.  Linkage to smartphone. | Output of different system configurations as basis for discussion and shift/widening of research focus.  Easy data input by e.g. gamification.  Generic enough to also be used in five years time. | Visual output (graphics and videos).  Need to know how to judge outputs of tool.  Interactive on basis of GIS denoting places with case-studies. | NA | NA | NA | Users require explanatory videos and written guidance on the use and applicability of the tool. Differentiated complexity of use, but easy data input, visual output of the results as well as an understanding of how to interpret these results. | Easy data input and visual output that shows different system configuration possibilities. |
|  | **Accessibility** | Through having full functionality on offline/low band-width devices usable by (non-) experts through interactive design and appropriate visuals on an open source platform. | To all people from the sector without overwhelming them.  Simple and possibility for direct use of the tool is a pre. | Accessible to everyone, not only the experts.  Different entry levels. Open-source. | Structured information trees.  Accessible offline and on low-bandwidth devices for people from all backgrounds and languages.  Connection to app-based data collection.  Differing levels of complexity (beginner, advanced, expert) to get quick but also detailed results. | By different users ranging from community planners to engineering experts. | NA | NA | NA | Users require full functionality offline and on low bandwidth devices, accessible to everyone, not only experts by differentiated level of complexity and an interactive design. The software should be open source. | SaniChoice can be used by everyone, from the general public to experts. It is open source and provides an interactive design. |
|  | **Credibility** | By uptake of the tool in practice, provision of uncertainty estimations, unbiased option selection and complete outputs. (partial outputs question validity).  Funding from international organizations | By uptake of the tool in practice, knowing ex-ante what the outcome could be (and interpretation thereof), and it is used by peers.  Transparency, evidence and full documentation of decision options.  Support from large organizations | Through case studies where tools are implemented and mobile labs to deliver evidence.  Compilation of information coming from community itself.  Show publications, data validation by renowned institutes.  Creating confidence for consultants. | Through case studies where tool is applied and proven useful.  Continuously updated content to reflect newest technologies, insights, etc.  No black box, source-code should be accessible as well as a full scientific documentation.  Everybody knows it. | Provision of benchmark by linking to case studies so that people can see how well it works in practice. | NA | NA | NA | Users define credibility mostly by the uptake of the tool in practice which is showcased by case studies and the backing from large organizations and peers. Evidence and uncertainty of performance data is important as well as full transparency in use of data and algorithms and continuous updated information. | SANTIAGO is tested in practice and is now adopted into SaniChoice that enables the wider adoption, thereby contributing to its credibility. Further it has the backing from renowned organizations and algorithms and use of data are completely transparent. |
|  | **Desirability** | A global platform on (country specific) details of technologies, add-like option proposals in combination with checklists on the enabling environment, data and knowledge gaps.  Connection to other sectors (e.g. energy projections) to support investment decisions.  Templates for videos and posters. | Visualization of treatment processes and short video clips.  Drag and drop system builder.  Templates for reports and presentations. | Toolbox interpreting legislation, funding, upkeep, and construction of proposed technologies.  Recommendation of tools based on users situation.  Top-notch graphical outputs to be presented and adopted in reports. | Introduction for people new to the field.  Showing current opportunities by e.g. research questions and needs from practice and industry.  Decision support based on technology, politics, and legal frameworks.  Small learning modules.  Manuals that translate into different contexts. | Materials to teach, learn, and plan should be attractive.  Two-hour training programs for trainers.  The content should be nested and linked to Sustainable Development Goals.  Planning processes. | NA | NA | NA | Users require an overarching platform connecting different disciplines and creating understanding of its complexity by checklists, and toolboxes that focus on the enabling environment. Further it should host ready to use materials to be adopted in presentations, reports, videos and posters. | SaniChoice does not answer to these specific requirement. |
