# Supplementary material for: Closing Water and Nutrient Cycles in Urban Wastewater Management: How to Make an Academic Software Available to General Practice
Source: Circ Econ Sustain. 2021 Jul 15;1(3):1087–105. doi: 10.1007/s43615-021-00073-6 (PMC8679645; doi:10.1007/s43615-021-00073-6)
Supplement: Supplementary file 4 — (DOCX 500 kb) [file 43615_2021_73_MOESM4_ESM.docx]

Closing water and nutrient cycles in urban wastewater management: how to make an academic software available to general practice

Johann S. Schuur^a,b^ and Dorothee Spuhler^a^

(0000-0002-0924-2319, 0000-0002-1379-6146)

Corresponding author: Johann S. Schuur, [jschuur@ethz.ch](mailto:jschuur@ethz.ch)

*^a)^ Eawag, Swiss Federal Institute of Aquatic Science and Technology, 8600 Dübendorf, Switzerland.*

*^b)^ ETH, Swiss Federal Institute of Technology, Institute of Science, Technology and Policy, 8092 Zürich, Switzerland.*

Description of content: This file contains the tentative architecture of SaniChoice
